# Two-dimensional semiconducting covalent organic frameworks via condensation at arylmethyl carbon atoms

**DOI:** 10.1038/s41467-019-10504-6

**Published:** 2019-06-06

**Authors:** Shuai Bi, Can Yang, Wenbei Zhang, Junsong Xu, Lingmei Liu, Dongqing Wu, Xinchen Wang, Yu Han, Qifeng Liang, Fan Zhang

**Affiliations:** 1 School of Chemistry and Chemical Engineering, State Key Laboratory of Metal Matrix Composites, Shanghai Jiao Tong University, 200240 Shanghai, China; 2 State Key Laboratory of Photocatalysis on Energy and Environment, College of Chemistry, Fuzhou University, 350002 Fuzhou, China; 3 Advanced Membranes and Porous Materials Center, Physical Sciences and Engineering Division, King Abdullah University of Science and Technology (KAUST), Thuwal, 23955-6900 Saudi Arabia; 4 Department of Physics, Shaoxing University, 312000 Shaoxing, China

**Keywords:** Photocatalysis, Photocatalysis, Conjugated polymers

## Abstract

Construction of organic semiconducting materials with in-plane π-conjugated structures and robustness through carbon-carbon bond linkages, alternatively as organic graphene analogs, is extremely desired for powerfully optoelectrical conversion. However, the poor reversibility for *sp*^2^ carbon bond forming reactions makes them unavailable for building high crystalline well-defined organic structures through a self-healing process, such as covalent organic frameworks (COFs). Here we report a scalable solution-processing approach to synthesize a family of two-dimensional (2D) COFs with *trans*-disubstituted C = C linkages via condensation reaction at arylmethyl carbon atoms on the basis of 3,5-dicyano-2,4,6-trimethylpyridine and linear/trigonal aldehyde (i.e., 4,4″-diformyl-*p*-terphenyl, 4,4′-diformyl-1,1′-biphenyl, or 1,3,5-tris(4-formylphenyl)benzene) monomers. Such *sp*^2^ carbon-jointed-pyridinyl frameworks, featuring crystalline honeycomb-like structures with high surface areas, enable driving two half-reactions of water splitting separately under visible light irradiation, comparable to graphitic carbon nitride (g-C_3_N_4_) derivatives.

## Abstract (web summary)

Semiconducting covalent organic frameworks (COF) with conjugated structures are desired for optoelectric conversion but poor reversibility of bond forming reactions hampers the formation of crystalline structures. Here, the authors synthesise COFs with disubstituted C = C linkages which enable water splitting under visible light irradiation.

## Introduction

**Fig. 1:**
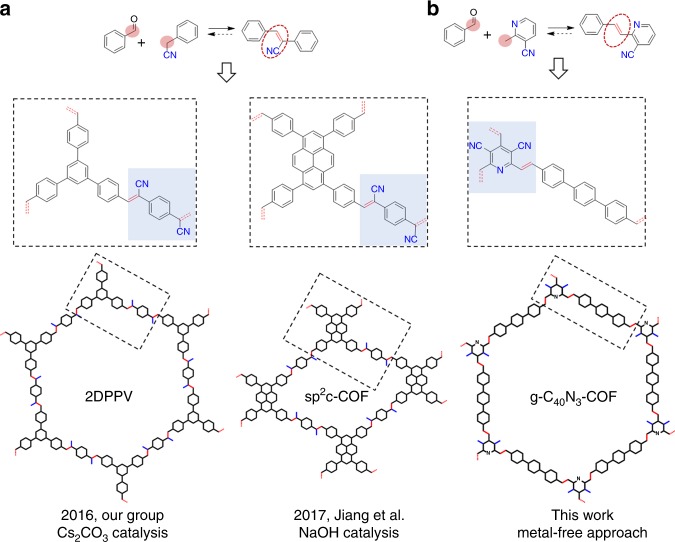
Chemical structures of the C = C bond linked covalent organic frameworks. **a** Representative two examples of 2D covalent organic frameworks with carbon–carbon double bond (C = C) linkages through Knoevenagel condensation at methylene carbon atoms by using inorganic base catalysts. **b** Metal-free approach to the target 2D covalent organic framework (g – C_40_N_3_-COF) with C = C linkages via condensation at arylmethyl carbon atoms upon organic base catalysis

Recently, 2D organic porous materials with well-defined π-extended structures, emerging as organic graphene analogs, have been successfully synthesized by connecting organic building blocks through carbon-carbon bond linkages^1–3^. They inherit the structural merits of graphene and π-conjugated organic molecules to some extent, promising for applications in optoelectronic and energy-storage devices^4,5^. Among these materials, a nano-porous graphene and a 2D conjugated aromatic polymer with carbon-carbon single bond (C – C) linkages have been synthesized through surface-mediated polymerization and solid-state topochemical polymerization, respectively^6,7^. In studies on these materials, rationally designed monomers for preassembly and precise regioselective coupling polymerization act as key roles in the formation of architectures with excellent topologies. In another case, we reported a 2D cyanostilbene-based covalent organic framework (COF) involving the connection of aromatic units through a carbon-carbon double bond (C = C) linkage under the Knoevenagel reaction (Fig. 1a)^8^. This report suggested that carbon-carbon bonding enables a system to undergo a thermodynamically controlled process, in which the crystallinity of an in-plane structure is governed through continued 2D chain growth and defect correction, as verified in the previously reported COFs with dynamic covalent linkages (e.g., imine)^9^. Afterwards, another 2D cyanostilbene-based COF was developed through a similar preparation protocol by using pyrene as one of the building blocks (Fig. 1a)^10^. Such kind of COFs exhibit π-delocalization over 2D networks, attributed to the efficient π-electron communication through C = C linkages, as verified by numerous C = C-containing artificial organic compounds and natural products (e.g., β-carotene) with unique photophysical properties. In a Knoevenagel reaction, the aryl α-carbon atom in a monomer can be easily converted to a reactive intermediate carbanion, which tends to be stabilized through a *p*-π-conjugated effect^11^. This carbanion can definitely promote the formation of a highly crystalline structure through self-healing processes. On the other hand, a C = C linkage enables avoiding the twisted conformations arising from steric repulsion in a main backbone when two aromatic units are directly coupled by a C – C single bond, thus beneficial to the formation of an in-plane π-conjugated structure. Nevertheless, it would be challenging to determine appropriate conditions or regulation for reversible C = C bond formation, even in the aforementioned Knoevenagel reactions, which, according to our knowledge, only succeeded for a few COF samples arising from 1,4-phenylenediacetonitrile (or its derivatives).

Owing to the highly exposed surface areas and tunable band gaps, polymeric semiconductors such as graphitic carbon nitride (g-C_3_N_4_) have been attracting considerable attention as high-performance photocatalysts for water splitting under visible light, which is one of the long-term goals for the efficient storage of intermittent solar energy^12–20^. The high crystallinity of 2D COFs has substantial advantages with respect to their well-defined structures, which can facilitate establishing structure-property relationships and provide insights into photocatalytic processes or mechanisms^21–24^. Attempts to promote the photocatalytic activities for COFs have mainly been hindered by their relatively low stability and poor π-electron delocalization^25,26^. Pyridine derivatives as nitrogen-embedded aromatic units feature Lewis basicity and versatile optoelectronic properties. In particular, completely different from the interrupted conjugation effect on the *meta*-positions of phenyl ring, the facile modification of pyridine in its 2,6-positions, 2,5-positions, or 2,4-positions enables achieving well extended π-conjugated systems, thus extensively used as building blocks or ligands for constructing organic or organometallic semiconductors with excellent photocatalytic behaviors^27,28^.

In this study, we utilize 3,5-dicyano-2,4,6-trimethylpyridine (DCTMP) as the key building block, whose three methyl carbon atoms are readily converted to relatively stable carbanions under an organic base catalyst in a p-π-conjugated system^29,30^. Upon condensation with 4,4″-diformyl-*p*-terphenyl (DFPTP), 4,4′-diformyl-1,1′-biphenyl (DFBP) and 1,3,5-tris(4-formylphenyl)benzene (TFPB) in N,N-dimethylformamide (DMF), three 2D COFs (denoted as g-C_x_N_y_-COFs) with *trans*-disubstituted C = C bond linkages are formed in nearly quantitative yields, termed as g-C_40_N_3_-COF, g-C_31_N_3_-COF, and g-C_37_N_3_-COF, respectively. Powder X-ray diffraction (PXRD), high-resolution transmission electron microscopy (HRTEM), and surface area measurement clearly reveal the COFs have honeycomb-like crystalline porous structure with high surface areas. The appropriate energy levels of conduction and valence bands of g-C_40_N_3_-COF allow for driving two half-reactions of water splitting separately to generate hydrogen or oxygen under visible light irradiation.

## Results

### Design and synthesis of g-C_x_N_y_-COFs

The target COFs were solvothermally synthesized through Knoevenagel condensation reaction between 3,5-dicyano-2,4,6-trimethylpyridine **(**DCTMP**)** and linear/trigonal aldehyde (4,4″-diformyl-*p*-terphenyl, 4,4′-diformyl-1,1′-biphenyl or 1,3,5-tris(4-formylphenyl)benzene) in DMF, catalyzed by the organic base piperidine. In this process, first, the pyridinyl methyl carbon atoms in DCTMP are converted to carbanions through C–H cleavage upon treatment with the base. This conversion is typically attributed to a decline in the electronic cloud density around these arylmethyl carbon atoms, which is caused by the strong electron-deficient pyridine ring that bears two electron-withdrawing cyano groups. The resulted carbanions tend to be stabilized through a strong *p*-π-conjugated interaction with this substituted pyridine ring, enabling the subsequent nucleophilic attacks on the positive carbon atoms in the aromatic formyl groups of these aldehydes. Such attacks form carbon-carbon bonds, which then transform to carbon–carbon double bonds by releasing H_2_O molecules. Under the guidance of such a reaction mechanism, we established an appropriate condition for achieving high-crystalline olefin-linked COFs. For example, in an optimized procedure, DCTMP (0.50 mmol), DFPTP (0.75 mmol), and piperidine (3.00 mmol) were dissolved in DMF (10 mL) and employed in a 15-mL pressure flask. The mixture was heated at 150 °C for 72 h, after which the resulted yellow solid was collected and purified through a standard workup to obtain the target COF g-C_40_N_3_-COF with nearly quantitative yield (see details in the methods section). In order for comparison, a model compound 3,5-dicyano-2,4,6-tristyrylpyridine (denoted as DCTSP) was also synthesized by a reaction of DCTMP and benzaldehyde under the same reaction condition.

### Characterizations of g-C_x_N_y_-COFs

**Fig. 2:**
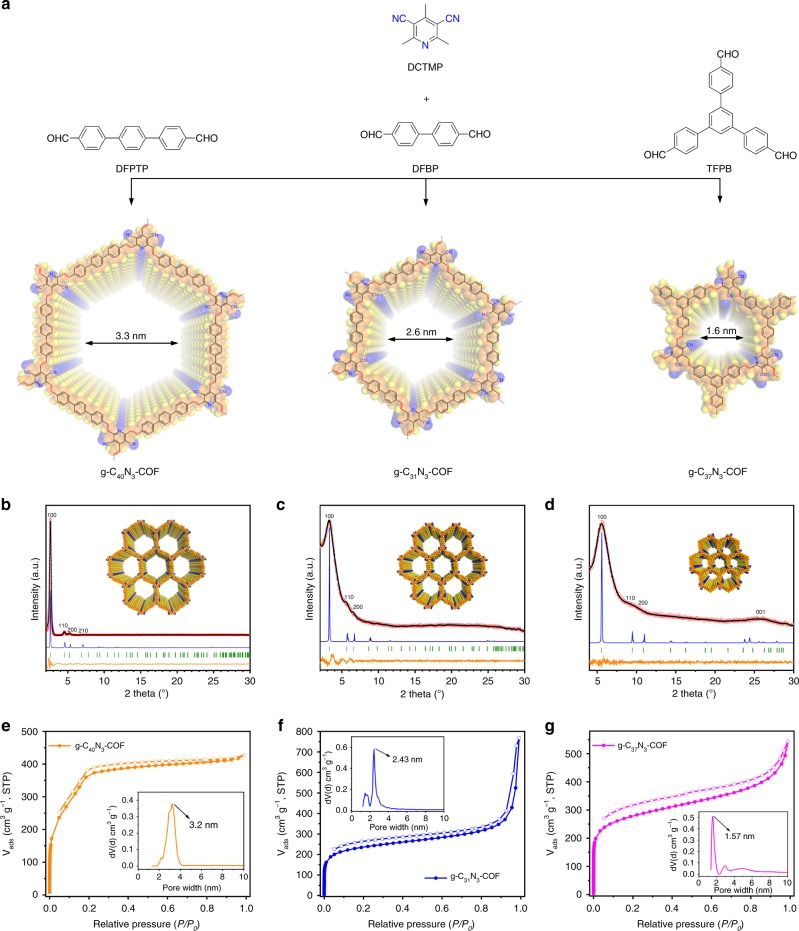
Characterizations of crystal and porous structures for g-C_x_N_y_-COFs. **a** Illustration of synthetic procedures of g-C_x_N_y_-COFs. **b**, **c** and **d** PXRD patterns for g-C_40_N_3_-COF, g-C_31_N_3_-COF, and g-C_37_N_3_-COF: comparison between the experimental (red circle) and Pawley refined (black) profiles, the simulated patterns for eclipsed AA stacking mode (blue), the Bragg positions (green) and the refinement differences (orange). Insets: the crystal structures of corresponding g-C_x_N_y_-COFs assuming the eclipsed stacking viewed along [001] directions. **e**, **f** and **g** Nitrogen adsorption and desorption isotherms of g-C_40_N_3_-COF, g-C_31_N_3_-COF, and g-C_37_N_3_-COF. Insets: the pore size distributions calculated from non-local density functional theory

PXRD analyses revealed the crystallinity of the resulting COFs. In conjunction with structural simulation, the structural features of g-C_x_N_y_-COFs were elucidated. Of these, the obtained PXRD pattern of g-C_40_N_3_-COF was dominated by an intense reflection in the low-angle region at 2*θ* = 2.62°, which was assigned to the (100) facet (Fig. 2b). Four other peaks at 4.53°, 5.23°, 6.94°, and 26.67° were assigned to the (110), (200), (210), and (001) facets, respectively (Supplementary Fig. 14). Fully eclipsed AA layer stacking and staggered AB layer stacking models were generated. Geometrical energy minimizations of the structural models were conducted using the Materials Studio software package and diffraction patterns were simulated. The experimental PXRD pattern agreed well with a pattern simulated from an AA-eclipsed layer stacking model with unit cell parameters (*a* = 37.419 Å, *b* = 37.843 Å, *c* = 3.588 Å and *α* = 90.044°, *β* = 90.061°, *γ* = 119.756°) in the space group ${\it{P}}\bar 1$. While, the staggered AB layer stacking model did not match the experimental pattern. Finally, Pawley refinement against experimental PXRD data provided good agreement factors (*R*_wp_ = 3.47% and *R*_p_ = 2.75%). In addition, the (100) peak had a small full-width at half-maximum value of 0.36°, which is comparable to those of imine-based COFs, indicating the high crystallinity of the framework. As contrast, g-C_31_N_3_-COF and g-C_37_N_3_-COF exhibited lower crystallinity, as demonstrated by their broadened diffraction peaks of (100) facets at 2*θ* = 3.36° and 5.61°. Simulated AA stacking modes matched better with their PXRD patterns, from which hexagonal pore diameters were estimated as 2.6 nm and 1.6 nm for g-C_31_N_3_-COF and g-C_37_N_3_-COF, respectively (Fig. 2c, d).

The permanent porosities of g-C_x_N_y_-COFs were investigated by nitrogen sorption analysis at 77 K. As illustrated in Fig. 2e, f, the nitrogen sorption isotherms of g-C_40_N_3_-COF and g-C_31_N_3_-COF are assigned to type-IV reversible isotherms, indicative of their mesoporous characteristics. Whereas, g-C_37_N_3_-COF shows type-I reversible isotherm, suggesting its microporous structure (Fig. 2g). The Brunauer-Emmett-Teller (BET) surface areas of g-C_40_N_3_-COF, g-C_31_N_3_-COF, and g-C_37_N_3_-COF were calculated to be 1235 m^2^ g^-1^, 864 m^2^ g^-1^, and 1012 m^2^ g^-1^, respectively, which were much higher than those for the other C = C linked COFs reported to date (Fig. 1a)^8,10^. The pore size distribution (PSD) was evaluated with non-local density functional theory (NLDFT). The PSDs of g-C_x_N_y_-COFs exhibited maxima of 3.2 nm, 2.43 nm, and 1.57 nm, which were highly in line with the predicted values for AA eclipsed geometries of the frameworks.

**Fig. 3:**
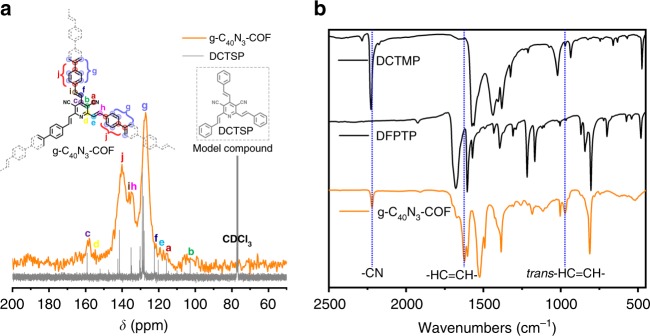
Characterizations of chemical structures for g-C_40_N_3_-COF. **a** ^13^C CP/MAS solid-state NMR spectra of g-C_40_N_3_-COF, compared with ^13^C NMR spectra of DCTSP (model compound) in CDCl_3_. **b** FT-IR spectra of g-C_40_N_3_-COF and corresponding monomers

The defined chemical structures of g-C_x_N_y_-COFs were confirmed through ^13^C cross-polarization/magic angle spinning solid-state nuclear magnetic resonance (CP/MAS ssNMR) spectroscopy, Fourier transform infrared (FT-IR) spectroscopy, thermogravimetric analysis (TGA), and elemental analysis. As an example, ^13^C ssNMR spectra of g-C_40_N_3_-COF and ^13^C NMR spectra of the model compound DCTSP in CDCl_3_ were combined in Fig. 3a. The peak at 117 ppm was assigned to the carbon atoms in cyano groups (a). The carbon atoms on pyridine unit (b, c, and d) corresponded to the peaks at 103, 159, and 155 ppm, respectively. The signals of vinyl carbon atoms (e, f, h and i) were found at 119, 120, 135, and 136 ppm, respectively. The intense resonance at 127 ppm was ascribed to the hydrogen-bonded aromatic carbons (g). The peak at 140 ppm was attributed to the carbon-attached aromatic carbons (j). FT-IR spectra (Fig. 3b) revealed that the stretching vibration peak of the cyano group (CN) was located at approximately 2225 cm^−1^, which was also observed for the monomer DCTMP. Moreover, a peak which was observed at 1690 cm^−1^ attributed to the C=O stretching vibration of the monomer DFPTP, disappeared in g-C_40_N_3_-COF, indicating a high polymerization degree. Notably, in addition to the peak at 1621 cm^−1^, which was attributable to the C = C moiety, the peak at 970 cm^−1^ engendered by the *trans*-HC = CH stretch vibration clearly manifested the *trans-*configurations of disubstituted olefin linkages in the entire framework. TGA revealed the extremely high thermal stability of g-C_40_N_3_-COF in N_2_ up to 500 °C, with the framework exhibiting less than 10% weight loss (Supplementary Fig. 6). Elemental analysis showed that the elemental contents (C: 84.68%, N: 8.38% and H: 4.95%) were close to the theoretical data (C: 87.89%, N: 7.69%, and H: 4.43%). X-ray photoelectron spectroscopy (XPS) further provided detailed information regarding the chemical structure of the framework (Supplementary Fig. 4). In high-resolution spectra, the C 1 *s* peak at 284.5 eV and the N 1 *s* peak at 399.0 eV were assigned to the *sp* hybrid C≡N moiety in cyano group. The peaks at 286.3 and 285.0 eV in the C 1 *s* zone were attributed to electrons originating from *sp*^2^ carbon atoms in vinyl groups and aromatic rings, respectively. The peak at 399.5 eV in the N 1 *s* zone was ascribed to pyridine nitrogen atoms. Similarly, the structural characterizations of g-C_31_N_3_-COF and g-C_37_N_3_-COF were also systematically performed for the verification of their chemical structures (Supplementary Figs. 1–6).

**Fig. 4:**
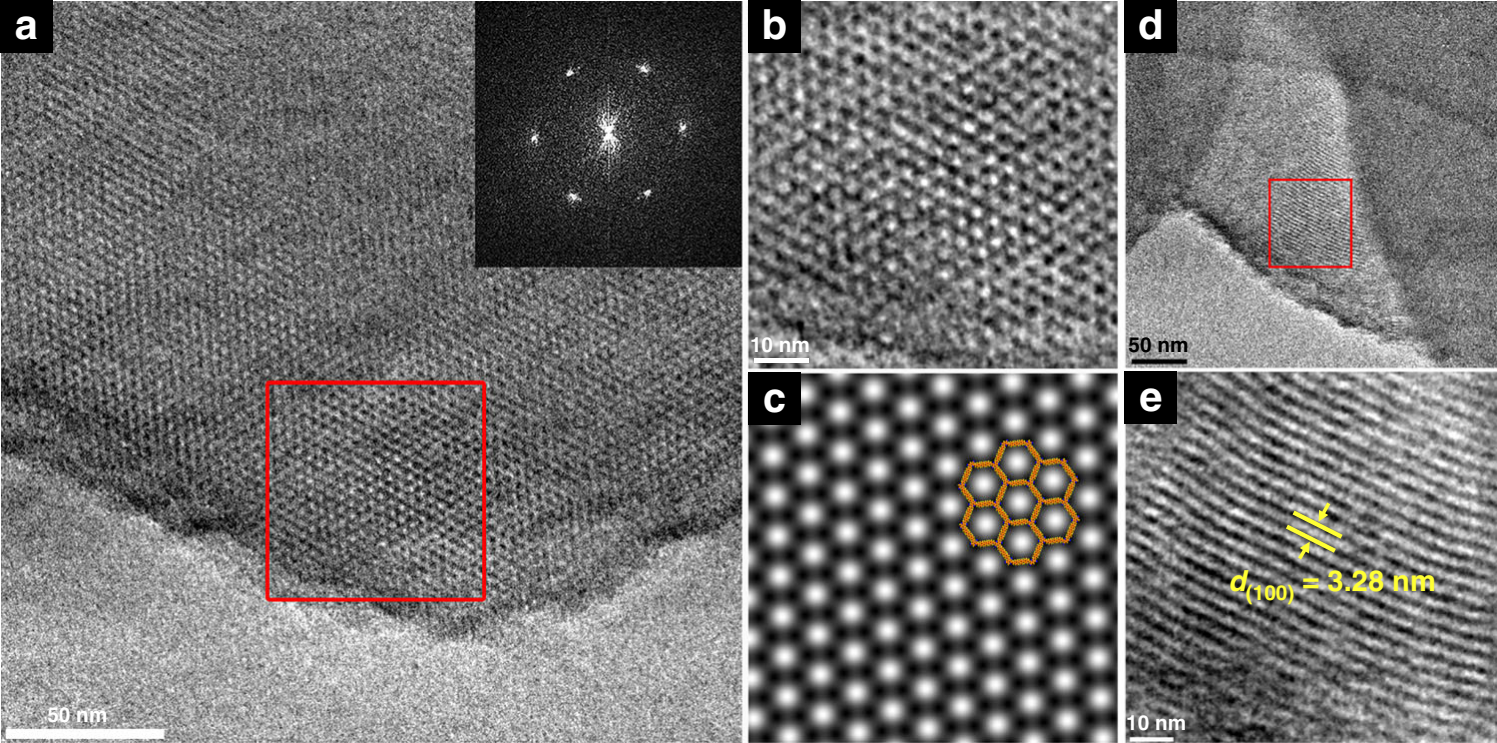
TEM characterization of g-C_40_N_3_-COF. **a** TEM image of g-C_40_N_3_-COF. Inset: fast Fourier transform (FFT) from the red square marked area. **b** high-resolution TEM image of g-C_40_N_3_-COF showing hexagonal pores viewing from [001] direction. **c** Simulated HRTEM image. The eclipsed structure model of g-C_40_N_3_-COF is overlaid. **d** TEM image of g-C_40_N_3_-COF from different areas. **e** Enlarged HRTEM image of red square marked area in **d**, showing 1D channels viewing from [110] direction with the pore channel diameter determined as 3.28 nm

HRTEM was used to further confirm the formation of a periodic porous framework for g-C_40_N_3_-COF. The lattice fringes are depicted in Fig. 4a, revealing an ordered alignment with a high degree of crystallinity. A honeycomb-like porous structure was clearly visualized along the [001] plane under high resolution (Fig. 4b). As indicated by a fast Fourier transform (inset in Fig. 4a) conducted on a selected area (red square in Fig. 4a), a hexagonal arrangement of six white diffraction spots was found in the raw HRTEM image, corresponding to (100) facet. An observation from the [110] direction (Fig. 4d) revealed the distinct one-dimensional channels with a uniform diameter of ~3.28 nm (Fig. 4e), which is consistent with the pore diameter of 3.3 nm in the simulated COF structure, according to PXRD analysis. In most cases of COFs, limited HRTEM images were achieved, primarily because skeletal structures could be easily damaged under high-energy electron beams. Recently, a low-dose TEM technique was developed for realizing well-resolved images of COFs with electron beam-sensitive linkages (e.g., imine bonds)^31^. By contrast, g-C_40_N_3_-COF could be clearly visualized even under normal TEM measurement conditions without any additional techniques. This phenomenon strongly supports the robust network of the as-synthesized COF through C = C linkages. Clear observation of ordered domains in the TEM images of g-C_31_N_3_-COF and g-C_37_N_3_-COF was difficult, likely due to their lower crystallinity than g-C_40_N_3_-COF (Supplementary Fig. 17).

### Optical and electronic properties

**Fig. 5:**
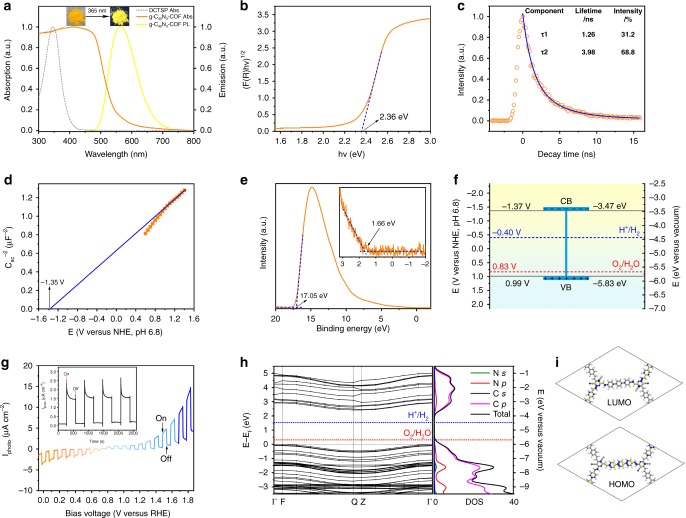
Optical and electronic properties of g-C_40_N_3_-COF. **a** UV/vis diffuse reflectance spectra (DRS) and steady-state photoluminescence (PL) spectra of g-C_40_N_3_-COF. The gray dash line represents solution UV/vis absorption spectra of DCTSP (model compound) recorded in dichloromethane. The upper inset shows the digital photograph of the sample under ambient light and 365 nm UV-lamp. **b** Band gap determined from the Kubelka–Munk-transformed reflectance spectra. **c** PL decay spectra monitored at 563 nm under 365 nm excitation at 298 K. **d** Mott-Schottky (M-S) plot for g-C_40_N_3_-COF measured in 0.2 M Na_2_SO_4_ (pH 6.8) with Ag/AgCl (+0.199 V vs. NHE) as the reference electrode in dark. **e** UPS spectrum of g-C_40_N_3_-COF. **f** Band positions of g-C_40_N_3_-COF (These vacuum level values were converted to electrochemical potentials according to: −4.44 eV vs. vacuum level is equal to −0.4 V vs. NHE at pH 6.8). **g** Chopped current–potential (I–V) curve of g-C_40_N_3_-COF photoelectrode under visible light (300 W Xenon light with cut-off filter λ > 420 nm). Electrolyte: Ar-saturated 0.2 M Na_2_SO_4_ aqueous solution. The applied potential was swept at +10 mV s^−1^ under intermittent irradiation with a period of 5 s. Inset is chopped anodic photocurrent density vs. time recorded on a g-C_40_N_3_-COF photoanode at 1.0 V vs. RHE. **h** Electronic properties of g-C_40_N_3_-COF using DFT calculations and corresponding density of states (DOS). **i** The Kohn-Sham orbitals of HOMO and LUMO of g-C_40_N_3_-COF. C gray, N blue, H white

The electronic properties of these g-C_x_N_y_-COFs were systematically investigated by photophysical and electrochemical measurements. We took the high crystalline COF g-C_40_N_3_-COF as an example for detailed elucidation. Its ultraviolet/visible diffuse reflectance spectroscopy (UV/vis DRS) showed a broad absorption band covering both UV and visible-light regions, with absorption edge at 550 nm (Fig. 5a). The absorption maxima at 500 nm for g-C_40_N_3_-COF was observed, which was red-shifted over 150 nm in comparison with that of 350 nm for DCTSP (model compound), indicating that the 2D network in the former case possesses extended π-conjugated structure. g-C_40_N_3_-COF with the longest polyphenylene spacer showed a redshift of the absorption edge in its DRS as compared to the other two COFs g-C_31_N_3_-COF and g-C_37_N_3_-COF, suggesting its stronger light harvesting ability in the visible region (Supplementary Fig. 7). Accordingly, the optical band gaps of g-C_x_N_y_-COFs were determined from the Kubelka-Munk (K-M) function. An optical band gap of 2.36 eV was determined for g-C_40_N_3_-COF (Fig. 5b), which was smaller than that of g-C_31_N_3_-COF (2.40 eV) and g-C_37_N_3_-COF (2.52 eV). Upon irradiation at 365 nm by UV lamp, the solid powder sample of g-C_40_N_3_-COF emitted strong orange-yellow fluorescence, its fluorescence spectra showed an emission maximum at 563 nm (Fig. 5a). This phenomenon was presumably attributed to that the chromophores fixed in the rigid framework can efficiently get rid of aggregate-caused emission quenching^32^. Similar fluorescent characters were also found for g-C_31_N_3_-COF and g-C_37_N_3_-COF (Supplementary Fig. 8a). Time-resolved fluorescence decay spectroscopy was performed to evaluate the extent of exciton recombination, and which provided information about the average lifetime of photo-excited electrons. The fluorescence decay curves of all three COFs fitted the lifetimes with two exponential components (Fig. 5c and Supplementary Fig. 8b), and the average lifetimes of g-C_40_N_3_-COF, g-C_31_N_3_-COF, and g-C_37_N_3_-COF were estimated to be 3.31, 2.71, and 2.58 ns, respectively. The longest fluorescence lifetime of g-C_40_N_3_-COF suggests its extended π-conjugated structure, favorable for charge isolation. To further study the electronic structure of g-C_40_N_3_-COF, Mott–Schottky (M-S) measurement was conducted to estimate its relative band positions. The positive slope indicates typical n-type semiconductor characteristic for g-C_40_N_3_-COF (Fig. 5d). The flat-band potential (E_fb_) of g-C_40_N_3_-COF was fitted to be −1.35 V vs. normal hydrogen electrode (NHE) at pH 6.8 from the *x* intercept of the liners region of M-S plots. Moreover, ultraviolet photoelectron spectroscopy (UPS) was used to determine the energy level of valence band maximum (E_VB_). The E_VB_ of −5.83 eV (vs. vacuum level) was calculated by subtracting the UPS width from excitation energy (He I, 21.22 eV) (Fig. 5e). Combined with the aforementioned optical band gap, the conduction band minimum (E_CB_) could be calculated as −3.47 eV vs. vacuum level (i.e., −1.37 V vs. NHE at pH 6.8) (Fig. 5f)^33^, which is quite similar to the above flat-band potential. For comparison, all data for these COFs were collected in Table 1. In addition, we performed density-functional-theory (DFT) calculations to theoretically confirm the electronic structures of g-C_40_N_3_-COF. The band structures with an ideal infinite model along the high symmetry line from the *k*-points in the first Brillouin zone were demonstrated in Fig. 5h, suggesting an indirect band gap of 2.292 eV, which was in agreement with experimental value (2.36 eV). The partial density of states (PDOS), as well as the wave functions of both conduction band minimum (CBM) and valence band maximum (VBM) was analyzed as shown in Fig. 5h, which clearly revealed that the top of VBM and the bottom of CBM were mainly contributed by the 2*p* orbital of C atoms. Such results suggest that the intrinsic electronic properties of the as-prepared COF are arising from the well π-delocalization over the carbon-rich 2D skeleton (Fig. 5i). In order to evaluate the photoelectric responses of these COFs, we performed photocurrent tests by using their film electrodes on indium-tin oxide (ITO) substrates in a solution of 0.2 M Na_2_SO_4_ (pH 6.8). The chopped current-potential (I-V) curve of g-C_40_N_3_-COF photoelectrode is shown in Fig. 5g, revealing the remarkable photoresponses towards light on/off switching. A cathodic photocurrent was observed at the potentials below 0.8 V vs. RHE, and showing a saturated current density value of ca. 2.5 µA cm^−2^ below 0.4 V vs. RHE. Meanwhile, at bias voltage above 0.8 V vs. RHE, an increased anodic photocurrent was found, reaching ca. 10 µA cm^−2^ at 1.8 V vs. RHE. Such an ambipolar photoconduction mode for g-C_40_N_3_-COF manifests its capability of the generation and migration of either electrons or holes upon light irradiation with respect to its preferable band structure for overall water splitting. The similar phenomenon was also observed in a few polymeric photocatalysts (e.g., g-C_3_N_4_)^34^. In addition, we tested long-time photocurrent response for g-C_40_N_3_-COF at an applied bias of 1.0 V vs. RHE, apparent anodic photocurrent was also detected upon the chopped irradiation via light on/off switching (Fig. 5g inset). The photocurrent density of g-C_40_N_3_-COF was nearly constant within the mearsurement period of 2600 s, indicating its excellent stability under light irradiation^35^. The photophysical properties and electrochemical behaviors of g-C_40_N_3_-COF encouraged us to further explore its application on photocatalytic water splitting. As mentioned, its band gap is sufficiently large to overcome the theoretical endothermic characteristic in water-splitting processes (1.23 eV). In addition, the energy level for the reduction of H_2_O to H_2_ was observed to be below the E_CB_ of g-C_40_N_3_-COF, and the energy level for the oxidation of H_2_O to O_2_ was slightly above the E_VB_ of g-C_40_N_3_-COF. The predicted CBM and VBM with respect to the LUMO and HOMO energy levels are well positioned for overall water-splitting. Therefore, the appropriate positions of the energy band structures for this type of COF permit the efficient transfer of photogenerated electrons and holes, respectively, and theoretically make it a promising photocatalyst for overall water splitting.

**Table 1:** Electronic properties of g-C_x_N_y_-COFs

Samples	λ_abs_^a^ (nm)	Optical gap^b^ (eV)	λ_em_^c^ (nm)	τ_avg_^d^ (ns)	Quantum yield (%)	E_fb_^e^ (V)	E_VB_^f^ (eV)	E_CB_^f^ (eV)
g-C_40_N_3_-COF	550	2.36	563	3.31	0.7	−1.35	−5.83	−3.47
g-C_31_N_3_-COF	560	2.40	572	2.71	2.0	−0.94	−6.31	−3.91
g-C_37_N_3_-COF	525	2.52	570	2.58	3.2	−1.78	−5.54	−3.02

^a^UV-vis absorption edge

^b^Determined from the Kubelka–Munk-transformed reflectance spectra

^c^Fluorescence emission upon excitation at 365 nm

^d^Average fluorescence lifetime recorded upon excitation at λ_exc_ = 365 nm with a laser and observed at λ_em_ = 563, 572, and 570 nm for g-C_40_N_3_-COF, g-C_31_N_3_-COF, and g-C_37_N_3_-COF, respectively

^e^The flat-band potentials obtained from Mott–Schottky measurement are with respect to NHE at pH 6.8

^f^These values determined from UPS and optical gap are with respect to vacuum level

### Photocatalytic properties

**Fig. 6:**
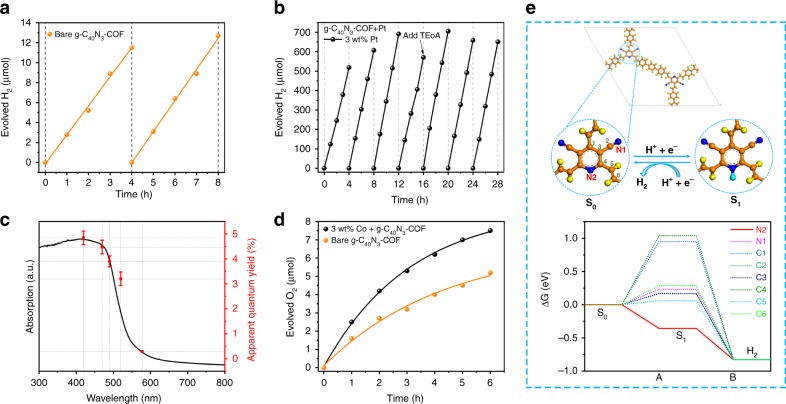
Photocatalytic hydrogen evolution, oxygen evolution, and AQYs of g-C_40_N_3_-COF. **a** Time course hydrogen evolution under visible light (λ > 420 nm) irradiation from H_2_O/TEoA (100 mL/10 mL) mixture using 50 mg bare (metal-free) g-C_40_N_3_-COF monitored over 8 h with evacuation every 4 h (dashed line). **b** Time course hydrogen evolution under visible light (λ > 420 nm) irradiation from H_2_O/TEoA (100 mL/10 mL) mixture using 50 mg of 3 wt% Pt modified g-C_40_N_3_-COF monitored over 28 h with evacuation every 4 h (dashed line). **c** Absorption spectrum and wavelength-specific apparent quantum yield (AQY) on H_2_ evolution measured with monochromatic LED light of wavelengths at 420, 470, 490, 520, and 578 nm, respectively, using 50 mg of 3 wt% Pt-modified g-C_40_N_3_-COF in H_2_O/TEoA (100 mL/10 mL) mixture. **d** Time course oxygen evolution from water containing 0.01 M silver nitrate as an electron acceptor under visible light (λ > 420 nm) by 3.0 wt% Co-loaded and unmodified g-C_40_N_3_-COF, respectively. La_2_O_3_ (0.2 g) was used as a buffer. **e** Top: reaction cycles and active sites for H_2_ evolution from water (C orange, N blue, H in COF: yellow, H from water: light blue). Bottom: free-energy variations for H_2_ evolution for each active site as labeled. The error bar represents the standard deviation from the repeated experiment after three times

H_2_ evolution experiments were performed by irradiating a suspension of 50 mg of COF in an aqueous solution containing sacrificial agent with visible light (λ > 420 nm) at 12 °C. After optimization of reaction conditions (Supplementary Figs. 34–36), 10 Vol% of triethanolamine TEoA (pH 10.30) was chose as sacrificial electron donor. Notably, in the absence of any cocatalyst (e.g., platinum), a bare sample of g-C_40_N_3_-COF enabled steady production of hydrogen with an average H_2_ production rate of 2.9 μmol h^−1^(Fig. 6a), which is comparable to the bulk g*-*C_3_N_4_, but rarely observed for other metal-free polymeric materials. Such a H_2_ production rate is still relatively low, mostly attributed to the sluggish hydrogen elimination from a hydrogenated COF surface^12^. Hence, different amount of platinum (Pt) was in situ deposited into the network of COF by irradiation of the suspension containing certain equivalents of hexachloroplatinic acid and COF samples in water and TEoA mixture for 4 h under visible light. This modification (deposition of Pt) enabled improving charge separation and facilitating proton reduction because of the lowest H_2_ absorption free energy for hydrogen release through the formation of Pt-H bonds. The correlation between the H_2_ production rate and the loading amount of Pt was systematically investigated. At the end of 4-hour in a series of tests, the average amount of hydrogen produced by 1, 2, 2.5, 3, 3.5, and 5 wt% Pt-modified g-C_40_N_3_-COF was 14.5, 22.3, 72.5, 129.8, 127.8 and 112.6μmol h^−1^, respectively (Supplementary Fig. 32). Thus, the 3 wt% Pt-modified g-C_40_N_3_-COF, which was the most active, was chosen for stability evaluation by running the reaction for a total of 28 h recorded in a 4-h interval under visible-light irradiation (λ > 420 nm) (Fig. 6b). The total amount of hydrogen evolved after 28 h was approximately 4400 μmol, which far exceeded the hydrogen generation rates of most previously reported COF-based photocatalysts and confirmed that the primary hydrogen source was water rather than the decomposition products of the COF^22^. During the long-term photocatalytic test, we found that the H_2_ evolution amount per hour increased stepwise in the first 4 h, after which it came to a plateau at an average H_2_ production rate of 153μmol h^−1^. After 16 h, the H_2_ evolution rate decreased slightly, probably due to the consumption of the sacrificial electron donor (i.e., TEoA). After adding another dose of TEoA, the highest H_2_ production rate of 206 μmol h^−1^ was achieved. The AQY is a crucial measure for evaluating the apparent efficiency of energy transformation from solar to hydrogen by photocatalysts, which is measured under monochromatic incident light. The AQY is calculated by dividing reactive electrons by the total incident photons and this value can be a standard for comparing the activities between different photocatalysts. In this study, the highest AQY obtained for g-C_40_N_3_-COF at 420 nm was 4.84% (±0.27%), which remarkably exceeded the AQYs of most promising polymer-based photocatalysts reported so far, such as a trazine-based N_3_-COF (AQY_420nm_ = 0.44%)^22^, a hydrazone-based TFPT-COF (AQY_400nm_ = 2.2%)^21^, an olefin-linked conjugated porous polymer OB-POP-3 (AQY_420nm_ =° 2.0%) in our previous work^36^, and sulfone-containing FS-COF (AQY_420nm_ = 3.2%)^37^. Moreover, the AQY recorded at 470, 490, 520, and 578 nm was 4.47% (±0.27%), 3.90% (±0.22%), 3.20% (±0.27%), and 0.29% (±0.01%), respectively (Fig. 6c). This phenomenon clearly proves that the light conversion efficiency is highly dependent on the light-harvesting level within the covered energy regions for g-C_40_N_3_-COF. And the fact also indicates that the process of H_2_ generation is indeed driven by the absorption of light. We characterized the molecular structure of g-C_40_N_3_-COF after long-term photocatalytic hydrogen evolution test by solid-state ^13^C NMR, FT-IR, UV/vis DRS, PXRD, and nitrogen sorption analysis, revealing its extremely high stability as a photocatalyst for the evolution of H_2_ from water reduction. Exceptionally, a slightly declined BET surface area was observed, which was possibly attributed to that the loading of Pt nanoparticles increased the mass of sample, as well as the pore channels could be blocked after long-time photocatalysis. In the TEM image of 3 wt% Pt loaded g-C_40_N_3_-COF after photocatalytic tests, the dendritic Pt nanoparticles (DPNs) were found with an average size of 22 nm (±2.7 nm) uniformly dispersed over the whole COF matrix (Supplementary Fig. 27a). Each DPN with branching in various directions was consisting of several domains with approximately 3 nm size, in which the lattice fringes with *d*-spacing of 0.23 nm with a dihedral angle of ∼70° and d-spacing of 0.2 nm with a dihedral angle of ∼90° were observed (Supplementary Fig. 28c, d), corresponding to the Pt {111} and Pt {100} facet, respectively. X-ray photoelectron spectroscopy (XPS) of the Pt loaded g-C_40_N_3_-COF sample was also recorded. The binding energy of electrons in pyridinyl nitrogen 1 *s* orbital was shifted from 399.5 eV to 399.8 eV, indicating that the coordination might occur between Pt and pyridinyl nitrogen atoms (Supplementary Fig. 27b). Pt 4 *f* core level XPS spectra could be deconvoluted into two pairs of peaks corresponding to Pt^0^ and Pt^2+^ on the basis of database values (Supplementary Fig. 27c). Pt^0^ was effective for H_2_ evolution while Pt^2+^ confirmed the coordination with pyridinyl nitrogen atom. Reasonably, the formation of such kinds of DPNs were highly associated with the presence of pyridine units in the network of g-C_40_N_3_-COF. A few DPNs had ever been achieved by using relatively complicated approaches(e.g., block polymer mediated synthesis)^38^, but seldom found for polymer-based photocatalysts (e.g., g-C_3_N_4_)^39^. The high surface areas of the resulting DPNs and their intensive interactions with the conjugated backbone of g-C_40_N_3_-COF were beneficial to enhancing photocatalytic activity. The intriguing properties and precise structural information of Pt-loaded g-C_40_N_3_-COF, might be available for the further development of ultrahigh performance catalytic systems under the reduced Pt consumption, such as single-atom Pt photocatalyst.

To gain more insights on the active sites of g-C_40_N_3_-COF for H_2_ evolution, the reaction process of proton-adsorption-reduction-hydrogen-adsorption was simulated using DFT calculations and the free-energy changes were calculated regarding to two types of nitrogen atom and six carbon atoms of g-C_40_N_3_-COF (Fig. 6e). The free-energy variations indicate that site N2 (pyridine-N) is favorable for single-site H_2_ evolution, implying that nitrogen atoms of pyridinyl rings in the network of g-C_40_N_3_-COF are the dominant active sites for photocatalytic H_2_ evolution, well in agreement with our previous discussion.

The other two COFs, g-C_31_N_3_-COF, and g-C_37_N_3_-COF were also investigated as photocatalysts for H_2_ evolution under the same reaction conditions. The preliminary results demonstrate that the H_2_ evolution rates for g-C_31_N_3_-COF and g-C_37_N_3_-COF are 27.1 and 19.8 μmol h^−1^, respectively, which are much lower than that of g-C_40_N_3_-COF (Supplementary Fig. 31). Apart from the light-harvesting and charge separation abilities, the crystallinity for these COF photocatalysts also serve as the crucial role in their photocatalytic activities for water-splitting, likely associated with the defects over the whole networks, as demonstrated by Cooper et al. very recently^37^.

Clearly, achieving an overall water-splitting process using artificial photocatalysts is the ultimate goal in the pursuit of renewable energy sources. Nevertheless, the water oxidation process can be sluggish, presumably due to low driving force and slow O-O bond formation kinetics, as observed for the representative metal-free photocatalyst g-C_3_N_4_^39,40^. In this study, the water oxidation performance of g-C_40_N_3_-COF was evaluated using AgNO_3_ as an electron acceptor and La_2_O_3_ as a pH buffer under visible-light irradiation. The unmodified g-C_40_N_3_-COF sample demonstrated a relatively low O_2_ production rate of 1.6 μmol h^−1^ (for 50 mg g-C_40_N_3_-COF). Loading cobalt species instead of noble metals into the COF samples as cocatalysts could engender a distinct performance enhancement. A low-cost non-noble metal salt Co(NO_3_)_2_, as the cobalt source (3 wt% Co^2+^), was introduced into the reaction system to modify the catalyst. As illustrated in Fig. 6d, the modified catalyst exhibited visible-light activity (λ > 420 nm) for O_2_ evolution with a rate of 2.5 μmol h^−1^. The resulting oxygen evolution rate is even better than that of bulk g-C_3_N_4_^12^. Although the O_2_ evolution activity of g-C_40_N_3_-COF was determined to be low, these results directly manifested the thermodynamic possibility of oxygen evolution as a reality. The low activity for O_2_ evolution might be ascribed to the smaller thermodynamic driving force of such type of COF as compared with that of H_2_ evolution, which could be further improved by rationally tuning semiconducting properties through precise structural modifications.

## Discussion

Typically, 2D COFs produced through C = C linkages possess unique geometric and electronic characteristics because they inherit notable advantages from conventional linear conjugated polymers and 2D graphene. The intriguing physical properties of these types of polymers render them highly desirable as semiconductors for optoelectronic applications. The traditional concept is that carbon-carbon bonds are irreversible and thus could not be used in the construction of highly crystalline structures through a thermodynamic process. Three years ago, we reported an example of a crystalline COF with cyano-substituted C = C linkages, which revealed the reversibility of C = C bonds in a Knoevenagel condensation reaction, allowing the formation of highly ordered structures through self-healing processes. In this study, we successfully constructed another family of 2D COFs linked by *trans*-disubstituted C = C bonds through condensation at arylmethyl carbon atoms. The highly crystalline frameworks were clearly revealed by PXRD, HRTEM, and surface area measurements. The frameworks’ intrinsic chemical structures, comprising pyridine core networks and neat C = C linkages, grant them π-electron delocalized properties and excellent light-harvesting characteristics. In addition, the appropriate band energy levels render the one of the framework g-C_40_N_3_-COF suitable as a photocatalyst for water splitting with two half-reactions in the presence of sacrificial reagents. A high hydrogen production rate was achieved, namely 206 µmol h^−1^ (relative to 50 mg of COF sample), with the apparent quantum efficiency of 4.84% at λ = 420 nm. This is among the highest values reported for COF-based photocatalysts so far. An oxygen evolution rate of 2.5 μmol h^−1^ was detected for COF photocatalyst when silver nitrate was applied as an electron acceptor under visible light, comparable to bulk g-C_3_N_4_. One can reasonably foresee that this work provides an approach to scalable and sustainable preparation of excellent 2D organic semiconductors or organic graphene analogs for potential practical applications far beyond photocatalysis.

## Methods

### Synthesis of g-C_x_N_y_-COF

All Chemicals were used as received without further purification. First, 0.50 mmol (85.60 mg) of 3,5-dicyano-2,4,6-trimethylpyridine (DCTMP) and 0.75 mmol (214.74 mg) of 4,4″-diformyl-p-terphenyl (DFPTP) (or 0.75 mmol (157.67 mg) of 4,4′-diformyl-1,1′-biphenyl (DFBP) or 0.50 mmol (195.22 mg) of 1,3,5-tris(4-formylphenyl)benzene (TFPB)) were placed in a 15-mL pressure flask. Then it was transferred into an argon-filled glovebox, where 3.00 mmol (255.45 mg) piperidine and 10 mL anhydrous deoxygenated N,N-dimethylformamide (DMF) were added. The mixture was stirred in glovebox for 5 min to dissolve the solid monomers into DMF. Then the pressure flask was sealed by Teflon nut and taken out from glovebox. Subsequently, the clear homogenous pale-yellow solution was heated in a 150 °C oil bath for 3 days. After cooling to room temperature, yellow precipitates were observed at the bottom of flask, leaving clear and colorless supernatant. The precipitate was filtered and washed by acetone and dichloromethane for several times, and then it was dried under vacuum for 12 h at 120 °C. Finally, yellow powder was obtained and the yield based on monomers was calculated to be approximately 99.3%.

### Photocatalytic hydrogen evolution

A flask was charged with 50 mg of g-C_40_N_3_-COF powder, 100 mL deionized water and 10 mL triethanolamine (TEoA). It was ultrasonicated for 15 min to obtain a well-dispersed suspension. Then the resulting suspension was transferred into a Pyrex top-irradiation reaction vessel connected to a closed gas system. Certain amount of platinum (Pt) as cocatalyst was loaded into the network of photocatalyst by in situ photodeposition method using H_2_PtCl_6_. The reaction mixture was evacuated several times to ensure complete removal of air prior to irradiation in a 90° angle with a 300 W Xe light-source. The wavelength of the incident light was controlled by using a 420 nm long pass cut-off filter. The temperature of the reaction solution was maintained at 12 °C by the flow of cooling water. The evolved gases were analyzed by gas chromatography equipped with a 5 Å molecular sieve column at 60 °C with argon as the carrier gas. Hydrogen was detected with a thermal conductivity detector (TCD) referencing against standard gas with a known concentration of hydrogen. Hydrogen dissolved in the reaction mixture was not measured and the pressure increase generated by the evolved hydrogen was neglected in the calculations. The hydrogen evolution rates were determined from a linear regression fit.

### Photocatalytic oxygen evolution

Fifty miligram of g-C_40_N_3_-COF was well dispersed by ultrasonication in an aqueous solution (100 mL) containing 0.01 mol L^−1^ AgNO_3_ as an electron acceptor and 0.2 g La_2_O_3_ as a pH buffer. The suspension was poured into a Pyrex top-irradiation reaction vessel connected to a closed gas system. Then it was evacuated several times to completely remove air prior to irradiation under a 300 W Xe lamp. The temperature of the reaction solution was maintained at 12 °C by the flow of cooling water. The evolved gases were analyzed by gas chromatography equipped with a 5 Å molecular sieve column at 60 °C with argon as the carrier gas. Oxygen was detected with a thermal conductivity detector (TCD) referencing against standard gas with a known concentration of oxygen.

### The apparent quantum yield (AQY) measurements

The apparent quantum yield (AQY) for H_2_ evolution was measured using monochromatic LED lamps with band pass filter of 420 ± 4.6 nm, 470 ± 4.4 nm, 490 ± 3.8 nm, 520 ± 4.0 nm, 578 ± 4.1 nm (errors of wavelength were depended on Guass Simulation of full width at half maximum), the intensities were 14.3, 11.2, 7.6, 2.9, 1.6 mW cm^−1^, respectively (ILT 950 spectroradiometer). The irradiation area was controlled as 3 × 3 cm^2^. Depending on the amount of hydrogen produced by the photocatalytic reaction in one hour, the AQY was calculated according to Eq. (1):

1 $$\begin{array}{c}{\mathrm{\eta }}_{{\mathrm{AQY}}} = \frac{{{\mathrm{N}}_{\mathrm{e}}}}{{{\mathrm{N}}_{\mathrm{p}}}} \times 100\% \\ = \frac{{2 \times {\it{M}} \times {\it{N}}_A}}{{\frac{{{\mathrm{E}}_{{\mathrm{total}}}}}{{{\mathrm{E}}_{{\mathrm{photon}}}}}}} \times 100\% \\ = \frac{{2 \times {\it{M}} \times {\it{N}}_A}}{{\frac{{{\it{S}} \times {\it{P}} \times {\it{t}}}}{{{\it{h}} \times \frac{{\it{c}}}{{\it{\lambda }}}}}}} \times 100\% \\ = \frac{{2 \times {\it{M}} \times {\it{N}}_A \times {\it{h}} \times {\it{c}}}}{{{\it{S}} \times {\it{P}} \times {\it{t}} \times {\it{\lambda }}}} \times 100\% \end{array}$$

Where, N_e_ is the amount of generated electrons, N_p_ is the incident photons, *M* is the amount of H_2_ molecules (mol), *N*_*A*_ is Avogadro constant (6.022 × 10^23^ mol^−1^), *h* is the Planck constant (6.626 × 10^-34^ J·s), *c* is the speed of light (3 × 10^8^ m s^−1^), *S* is the irradiation area (cm^2^), *P* is the intensity of irradiation light (W cm^−2^), *t* is the photoreaction time (s), *λ* is the wavelength of the monochromatic light (m).

## Supplementary information

Supplementary Information

## Data Availability

All data supporting the findings of this study are available within the article, as well as the Supplementary Information file, or available from the corresponding authors on reasonable request.
