# Supplementary material for: Two-dimensional semiconducting covalent organic frameworks via condensation at arylmethyl carbon atoms
Source: Nat Commun. 2019 Jun 6;10:2467. doi: 10.1038/s41467-019-10504-6 (PMC6554277; doi:10.1038/s41467-019-10504-6)
Supplement: Supplementary file 1 — Supplementary Information [file 41467_2019_10504_MOESM1_ESM.pdf]

Supplementary Information

Two-dimensional semiconducting covalent organic frameworks via condensation at arylmethyl carbon atoms

Bi et al.

Supplementary Methods.

Materials: 3,5-Dicyano-2,4,6-trimethylpyridine (**DCTMP**)[1], 4,4''-diformyl-p-terphenyl (**DFPTP**)[2] and 1,3,5-tris(4-formylphenyl)benzene (**TFPB**)[3] were prepared using reported methods. 4,4'-Diformyl-1,1'-biphenyl (**DFBP**) was purchased from Energy Chemical. Piperidine and anhydrous DMF were purchased from J&K. All the solvents were purchased from Adamas-beta Reagent and used as received without further purification.

General methods: The reactions involving inert atmosphere were carried out using standard schlenk technique or in an MBraun glovebox. Solution nuclear magnetic resonance (NMR) spectra were measured on Mercury plus 400 (400 MHz for proton, 100 MHz for carbon) spectrometer with tetramethylsilane as the internal reference using $CDCl_3$ as solvent in all cases. ^{13}C cross-polarization/magic angle spinning solid-state nuclear magnetic resonance (CP/MAS ssNMR) experiments were performed on a Bruker AVANCE III 400 WB spectrometer operating at 100.62 MHz for ^{13}C using a double resonance 4 mm MAS NMR probe and a sample spinning rate of 10 kHz. The cross polarization time was 1 ms. The chemical shifts were referenced with adamantane. UV–vis diffuse reflectance spectra (UV–vis DRS) were recorded at room temperature on Varian Cary 500 Scan UV–visible system. Photoluminescence (PL) spectra were obtained with a FluoroMax-4 spectrophotometer. Nitrogen physisorption analyses were performed at 77 K using Micromeritics ASAP 3020 equipment. Thermal gravimetric analyses (TGA) were performed on a Perkin-Elmer TGA-7 thermogravimetric analyzer in nitrogen atmosphere from ambient temperature to 800 °C at the rate of 10 °C min^{-1}. Fourier transform infrared (FT-IR) spectra were recorded with a Spectrum 100 spectrometer (Perkin Elmer, Spectrum 100). Powder X-ray diffraction patterns were recorded on a Bruker D8 Advance diffractometer with Cu–$K_{\alpha 1}$ radiation (λ = 1.5406 Å). X-ray photoelectron spectroscopy (XPS) experiments were carried out on an AXIS Ultra DLD system from Kratos with mono Al Kα radiation (1486.6 eV) as X-ray source, the C 1s value was set at 284.6 eV for charge corrections. Elemental analysis was carried out using elemental analyser (EA) on a Vario-EL Cube. SEM measurements were performed on a FEI Sirion-200 field emission scanning electron microscope. Transmission electron microscope (TEM) characterizations were conducted using a Talos F200X with an accelerating voltage of 200 KV. The ultraviolet photoelectron spectroscopy (UPS) spectra were measured using ESCALAB250Xi instrument with a monochromatic He I light source (21.22 eV).

Electrochemical measurements: Indium-tin oxide (ITO) glasses were firstly cleaned by sonication in acetone for 30 min and dried under nitrogen flow. 5 mg of COF powder was mixed with 2 mL *N*,*N*-dimethylformamide and ultra-sonicated for 30 min to get slurry. The slurry was spreading onto ITO glass whose boundary was previously protected using Scotch tape. After air drying, the Scotch tape was unstuck and the uncoated part of the electrode was isolated with epoxy resin. A conventional three electrodes cell was used with a platinum wire as the counter electrode and a Ag/AgCl electrode (saturated KCl) as reference electrode. The electrolyte was a 0.2 M Na_2SO_4 aqueous solution (pH 6.8) and was purged with argon gas for 1 h prior to the measurements. The working electrodes were immersed in the electrolyte for 60 s before any measurement was taken. The photocurrent measurements were conducted with a BAS Epsilon workstation, with the working electrodes irradiated from the back side in order to minimize the influence of thickness of the

semiconductor layer. The visible light was generated by a 300W xenon lamp (PerfectLight, PLS-SXE300/300UV) with a $\lambda > 420$ nm cut-off filter, and was chopped manually. For Mott-Schottky experiments, the perturbation signal were 10 mV with the frequency of 800 Hz. The applied potentials vs. Ag/AgCl is converted to NHE or RHE potentials using the following equations:

$$E_{NHE} = E_{Ag/AgCl} + E^{\theta}_{Ag/AgCl}\ (E^{\theta}_{Ag/AgCl} = 0.199\ V) \quad (1)$$

$$E_{RHE} = E_{Ag/AgCl} + 0.0591pH + E^{\theta}_{Ag/AgCl}\ (E^{\theta}_{Ag/AgCl} = 0.199\ V) \quad (2)$$

Synthetic procedures of 3,5-dicyano-2,4,6-tristyrylpyridine (model compound):

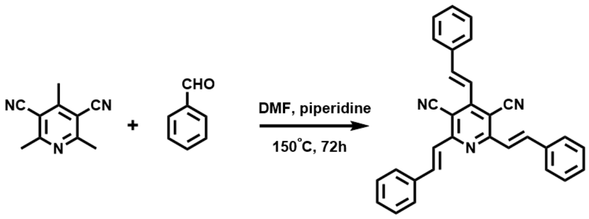

An oven-dried long-neck Schlenk flask was equipped with a magnetic stir bar and charged with 3,5-dicyano-2,4,6-trimethylpyridine (**DCTMP**) (85.6 mg, 0.5 mmol), benzaldehyde (318.5 mg, 3.0 mmol), piperidine (255.5 mg, 3.0 mmol) and 10 mL anhydrous DMF under the protection of nitrogen. Then the mixture was heated to 150 °C and stirred for 72 hours under nitrogen atmosphere. After cooling down to room temperature, the solution was poured into water and extracted with dichloromethane, dried over $MgSO_4$, and concentrated to obtain a brown solid. The crude product was purified by column chromatography over a silica gel column using petroleum ether–dichloromethane (v/v, 1/1) as the eluent to afford model compound as yellow solids. Yield: 52.8% (230.0 mg). ^{1}H NMR (400 MHz, $CDCl_3$): δ 8.28 (d, J = 15.4 Hz, 2H), 7.96 (d, J = 16.5 Hz, 1H), 7.64 (d, J = 15.4 Hz, 2H), 7.77-7.73 (m, 4H), 7.69-7.66 (m, 2H), 7.48-7.42 (m, 9H), 7.38 (d, J = 16.5 Hz, 1H); ^{13}C NMR (100 MHz, $CDCl_3$): δ 159.24, 152.10, 147.43, 142.36, 141.46, 135.22, 134.91, 130.47, 130.27, 129.88, 129.03, 128.99, 128.35, 127.98, 122.42, 120.29, 115.74, 102.80; HRMS (m/z): $[M+H]^+$ calcd. for $C_{31}H_{21}N_3$, 436.1735; found, 436.1833.

Structure simulations: Structural modeling of ***g*-C$_{40}$N$_3$-COF** was generated using the Accelrys Materials Studio software package. Prof. Yumei Zhang at the College of Materials Science and Engineering, Donghua University, China, allowed us to use their software package to carry out the simulations. Geometry optimization of fragment model was performed with DMol3 module. The initial lattice was created by starting with the space group of *P*3. The *a* and *b* lattice parameters (initially 37.7621 Å) were estimated according to the center to center distance between the vertices of the COF (**DCTMP** center to another **DCTMP** center). Then we degraded the symmetry of the lattice to *P*1, inserted the optimized fragment model in the empty cell and promoted the symmetry to $P\bar{1}$, outputting the crude structure of ***g*-C$_{40}$N$_3$-COF**. The lattice model was geometry optimized using the Forcite module (Universal force fields, Ewald summations). Finally, Pawley refinement was applied to define the lattice parameters, producing the refined PXRD profile, R_{wp} and R_p values. Staggered arrangements were also examined. Comparison of the observed and the simulated PXRD patterns suggested that the preferable structure of ***g*-C$_{40}$N$_3$-COF** are the eclipsed arrangement.

Presence of 3,5-dicyano-2,4,6-trimethylpyridine (**DCTMP**) units in the COF gives rise to conceivable lattice symmetry as space group of $P\bar{1}$ and *P*1, according to the configurations between two adjacent pyridyl vertices in the framework. Based on the energy calculations, compared with

staggered structure, the lower energy configuration of both $P\overline{1}$ and $P1$ unit cells adopt eclipsed structure. The eclipsed unit cell with higher symmetry space group of $P\overline{1}$ has a lower relative energy compared to the eclipsed unit cell with $P1$ space group (147.372005 vs. 147.678884 kcal/mol). Thus we presume that the unit cell of ***g*-$C_{40}N_3$-COF** adopts eclipsed AA-stacking model with $P\overline{1}$ space group.

Similar simulations were performed for ***g*-$C_{31}N_3$-COF** and ***g*-$C_{37}N_3$-COF**, respectively. The AA-stacking models seem fitting better with their PXRD patterns.

Computational methods: The band structure calculations were performed using density functional theory (DFT), as implemented in the DMol3 program based on Materials Studio 8.0. The electronic exchange correlation energy was described by the generalized gradient approximation (GGA) method with Perdew-Burke-Ernzerhof (PBE) functional. Valence orbitals were described with the double numerical plus polarization (DNP) basis. The corresponding band structure and density of states are shown in Fig. 5h.

The electronic structure calculation of ***g*-$C_{40}N_3$-COF** was further performed by first-principles calculations based on the density functional theory (DFT) method as implemented in Vienna *ab initio* simulation package (VASP). Geometry optimizations were performed using the Perdew−Burke−Ernzerhof (PBE) type of generalized gradient approximation (GGA) for exchange-correlation functionals. An energy cutoff of 400 eV was used for plane-wave basis expansion. The atomic positions were relaxed so that the force on each atom is less than 0.02 eV/Å and the energy of the structure is converged to less than 5×10^{-6} eV/atom. Hybrid functional of Heyd, Scuseria, and Ernzerhof (HSE06) were used for electronic structure calculations.

Photocatalytic H_2 evolution reaction of ***g*-$C_{40}N_3$-COF** was simulated with the model proposed by Nørskov et al.[8]. In aqueous solution, HER process could be decomposed into two one-electron steps **A** and **B** with each step consuming a proton and an electron. The two steps of HER process is summarized as below (step **A** to **B**):

$$\mathbf{A: S_0 + H^+ + e^- \rightarrow S_1} \tag{3}$$

$$\mathbf{B: S_1 + H^+ + e^- \rightarrow S_0 + H_2} \tag{4}$$

Where $\mathbf{S_1}$ denotes the intermediate of ***H** at surface.

Considering the effect of pH value of solvent and external potential **U**, the Gibbs free energy changes Δ**G** are obtained through the following calculations:

$$\mathbf{\Delta G_A = G_{S_1} - \frac{1}{2}G_{H_2} - G_{S_0} + \Delta pH - eU_{red}} \tag{5}$$

$$\mathbf{\Delta G_B = G_{S_0} + \frac{1}{2}G_{H_2} - G_{S_1} + \Delta pH - eU_{red}} \tag{6}$$

where the Gibbs free energy of $\mathbf{H^+ + e^-}$ has been replaced by at standard conditions of pressure and temperature. The $\mathbf{\Delta pH = 0.0594 \times pH}$ is used to estimate the effect of pH value of solvent. Furthermore, the Gibbs free energy is shifted by the applied reduction ($\mathbf{eU_{red}}$) potential. For HER, $\mathbf{U_{red}}$ is the energy of excited electron. According to the position of CBM in ***g*-$C_{40}N_3$-COF** calculated from DFT, the $\mathbf{U_{red}}$ is 0.91 eV.

Supplementary Figures.

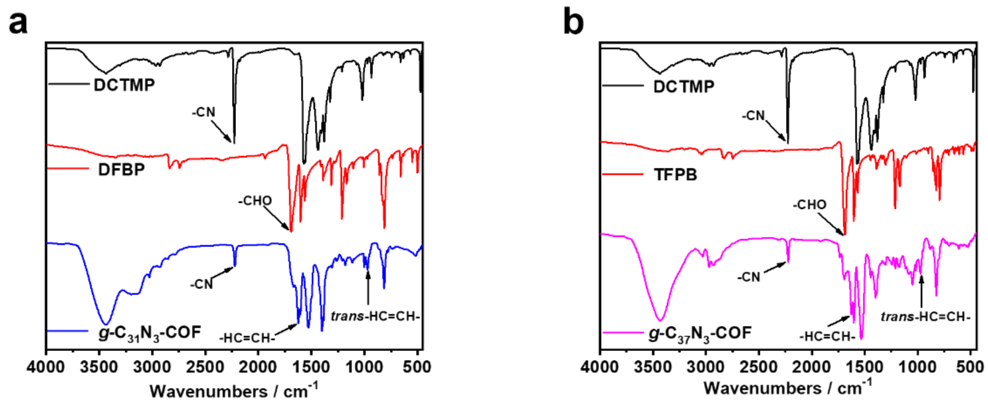

Supplementary Figure 1. FT-IR spectra of (a) ***g*-$C_{31}N_3$-COF**, (b) ***g*-$C_{37}N_3$-COF** and corresponding monomers.

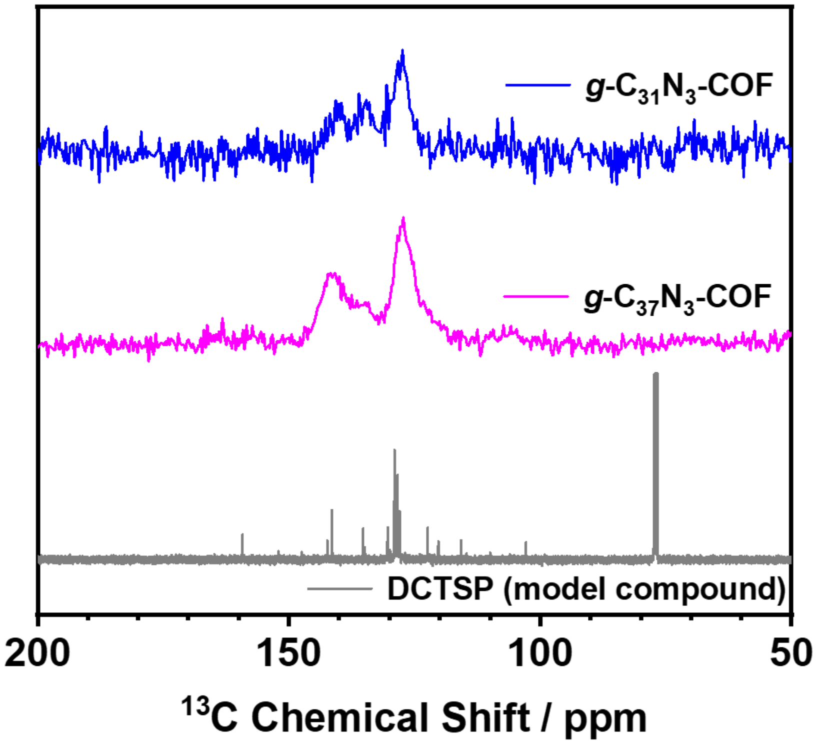

Supplementary Figure 2. ^{13}C CP/MAS solid-state NMR (ssNMR) spectra of ***g*-$C_{31}N_3$-COF**, ***g*-$C_{37}N_3$-COF** and **DCTSP** (model compound).

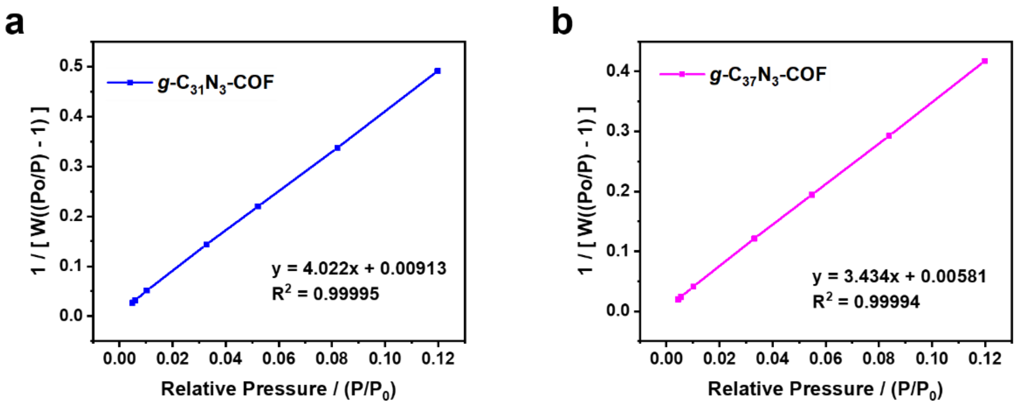

Supplementary Figure 3. Plots of the linear region for the BET equation of (a) ***g*-C$_{31}$N$_3$-COF** and (b) ***g*-C$_{37}$N$_3$-COF**.

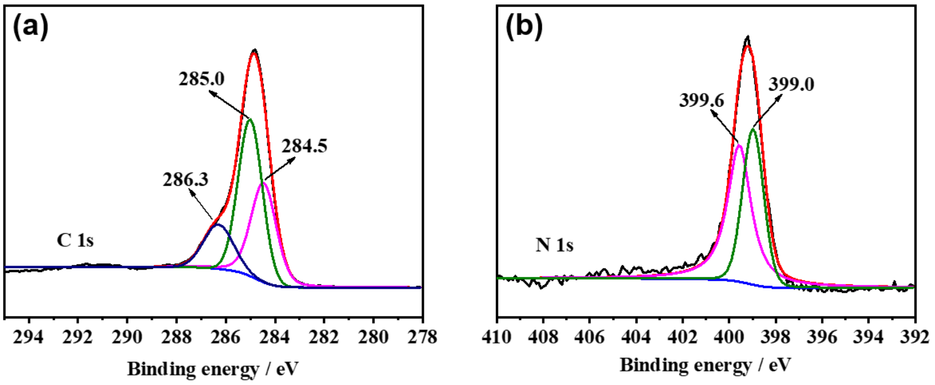

Supplementary Figure 4. X-ray photoelectron spectroscopy (XPS) spectra of C 1*s* (a) and N 1*s* (b) of ***g*-C$_{40}$N$_3$-COF**.

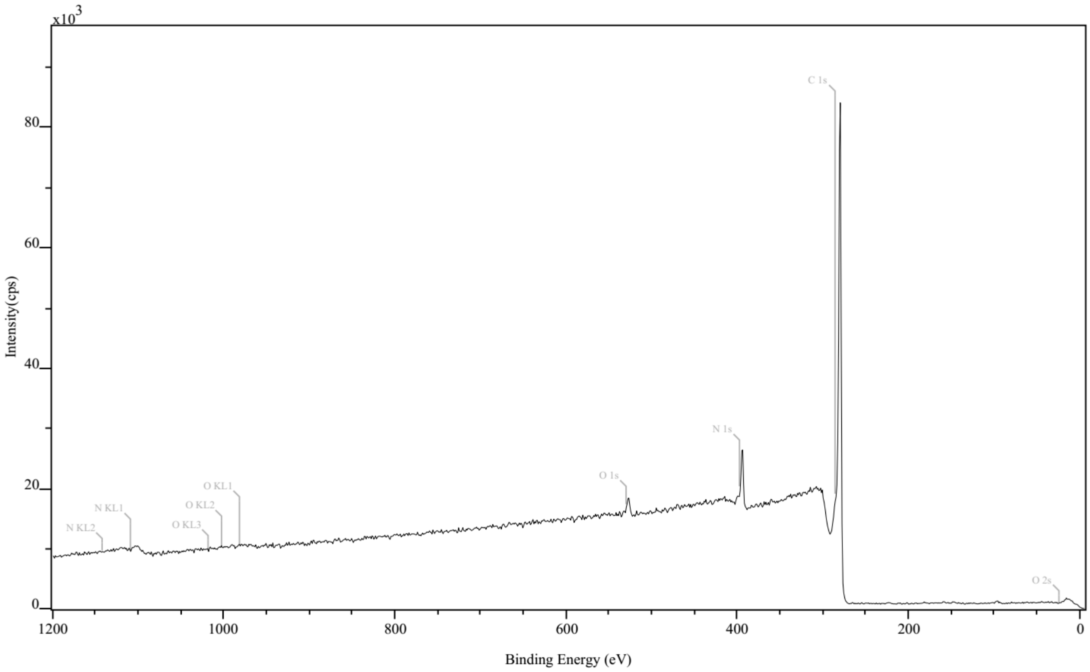

Supplementary Figure 5. XPS survey spectrum of ***g*-C$_{40}$N$_3$-COF**.

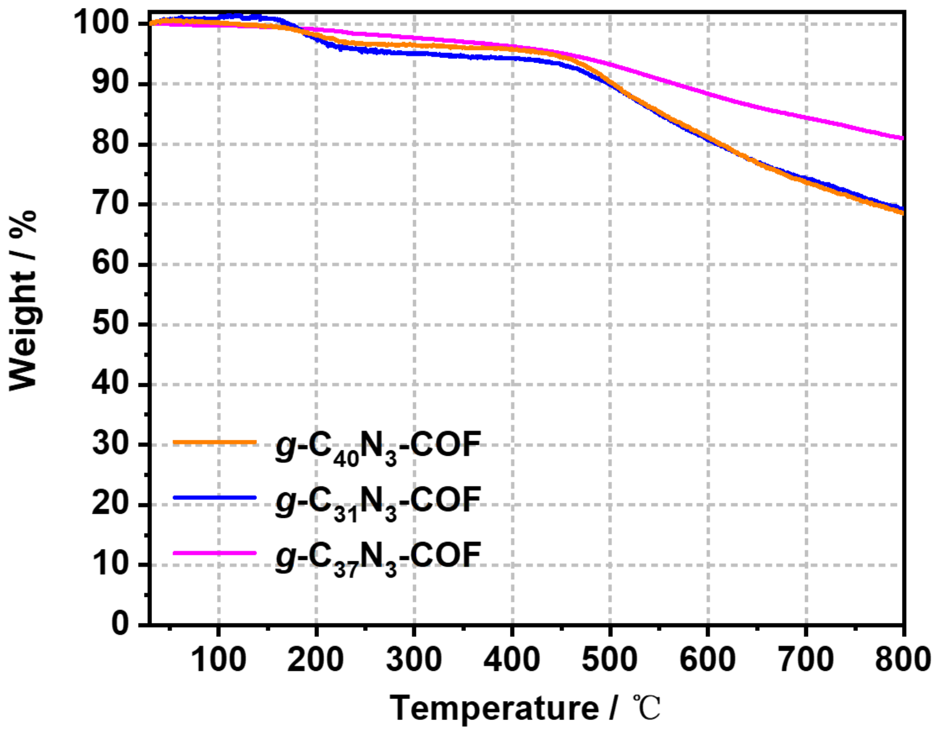

Supplementary Figure 6. Thermal gravimetric analysis (TGA) profiles of ***g*-$C_{40}N_3$-COF**, ***g*-$C_{31}N_3$-COF** and ***g*-$C_{37}N_3$-COF**.

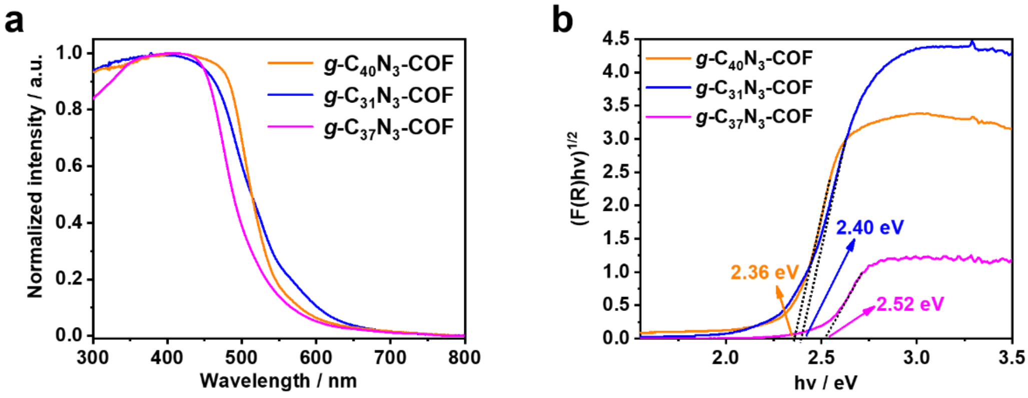

Supplementary Figure 7. (a) UV-vis DRS of ***g*-$C_{40}N_3$-COF**, ***g*-$C_{31}N_3$-COF** and ***g*-$C_{37}N_3$-COF**. (b) The corresponding band gaps determined from the Kubelka–Munk–transformed reflectance spectra.

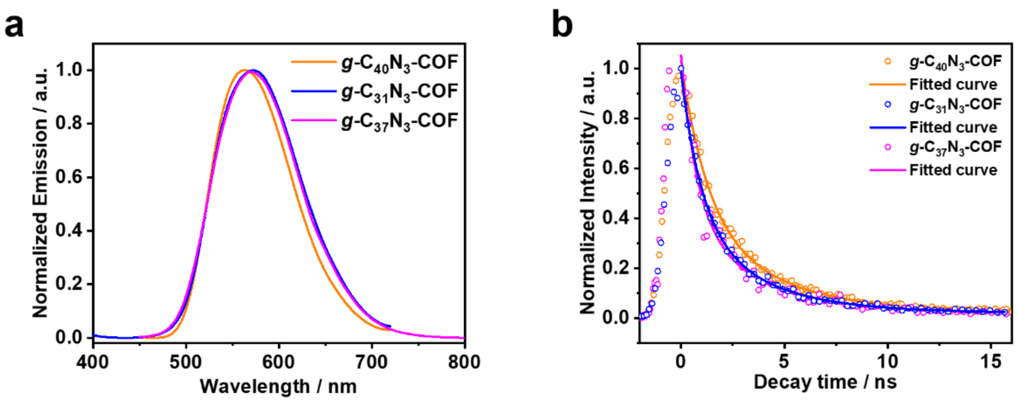

Supplementary Figure 8. (a) steady–state photoluminescence (PL) spectra of ***g*-C$_{40}$N$_3$-COF**, ***g*-C$_{31}$N$_3$-COF** and ***g*-C$_{37}$N$_3$-COF**. (b) The corresponding PL decay spectra.

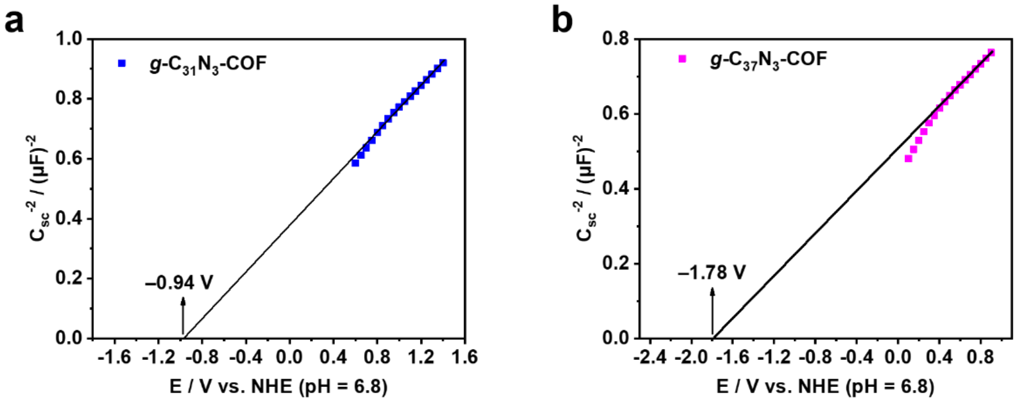

Supplementary Figure 9. Mott-Schottky plots of (a) ***g*-C$_{31}$N$_3$-COF** and (b) ***g*-C$_{37}$N$_3$-COF** measured in 0.2 M Na_2SO_4 (pH 6.8) with Ag/AgCl (+ 0.199 V vs. NHE) as the reference electrode in dark at 800 Hz.

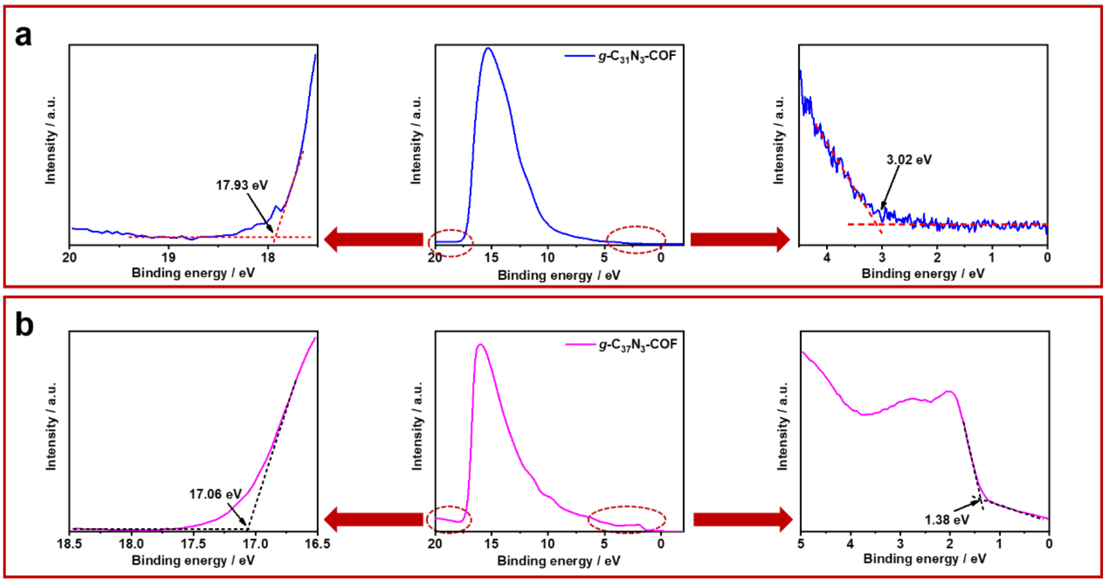

Supplementary Figure 10. UPS spectra of (a) ***g*-C$_{31}$N$_3$-COF** and (b) ***g*-C$_{37}$N$_3$-COF**.

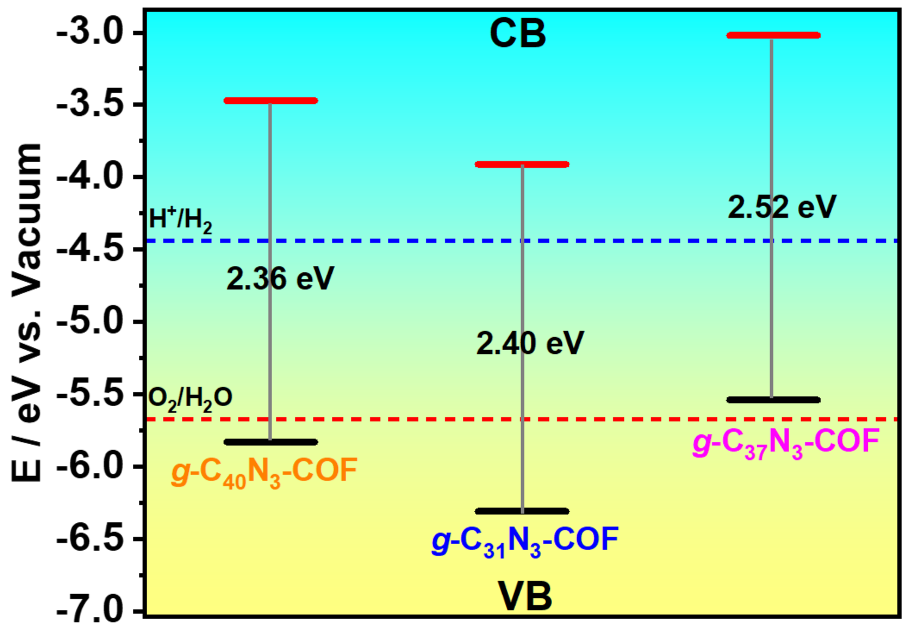

Supplementary Figure 11. The band structures of ***g*-C$_{40}$N$_3$-COF**, ***g*-C$_{31}$N$_3$-COF** and ***g*-C$_{37}$N$_3$-COF** with respect to vacuum level, together with the reduction level for H^+ to H_2 as well as the oxidation potential of H_2O to O_2. The E_{VB} of the samples were determined from UPS spectra. The E_{CB} of the samples were determined from E_{VB} and optical band gap.

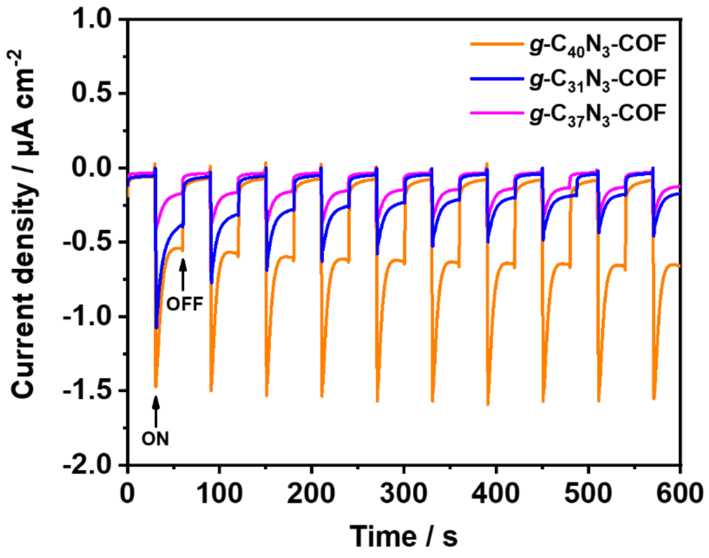

Supplementary Figure 12. Photocurrent densities *vs.* time for ***g*-C$_{40}$N$_3$-COF**, ***g*-C$_{31}$N$_3$-COF** and ***g*-C$_{37}$N$_3$-COF** with a bias of 0 V *vs.* Ag/AgCl (*i.e.* 0.6 V *vs.* RHE) under intermittent irradiation (measured in 0.2 M Na_2SO_4 aqueous solution, pH 6.8).

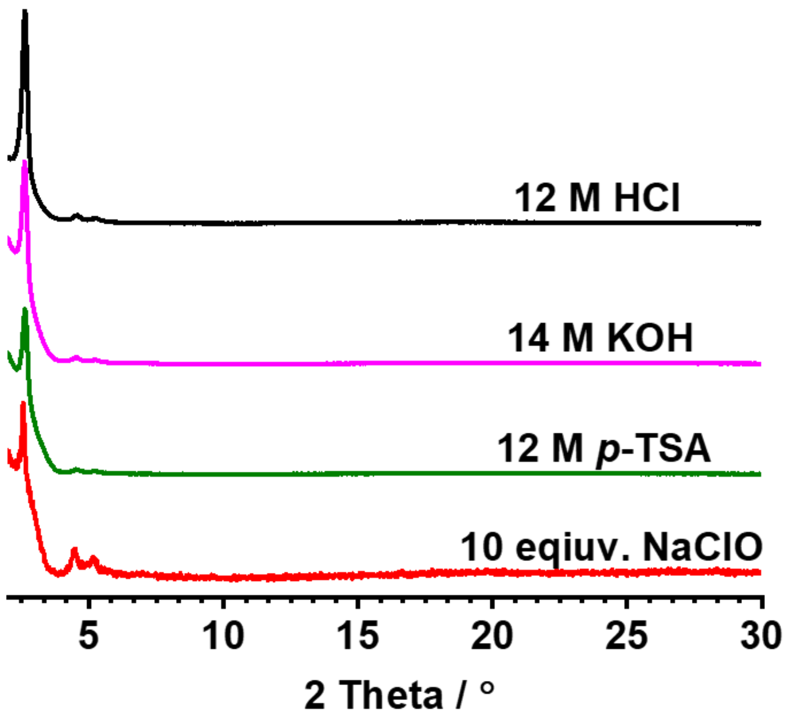

Supplementary Figure 13. Chemical stability of ***g*-C$_{40}$N$_3$-COF**. PXRD patterns of ***g*-C$_{40}$N$_3$-COF** after treatment with 12 M HCl (black), 14 M KOH in H_2O/MeOH (v/v, 1/1) solution (purple), 12 M *p*-toluenesulfonic acid (*p*-TSA) in H_2O/MeOH (v/v, 1/1) solution (green), and 10 equiv. of NaClO in H_2O/MeOH (v/v, 1/1) solution (red) at ambient temperature for 2 weeks.

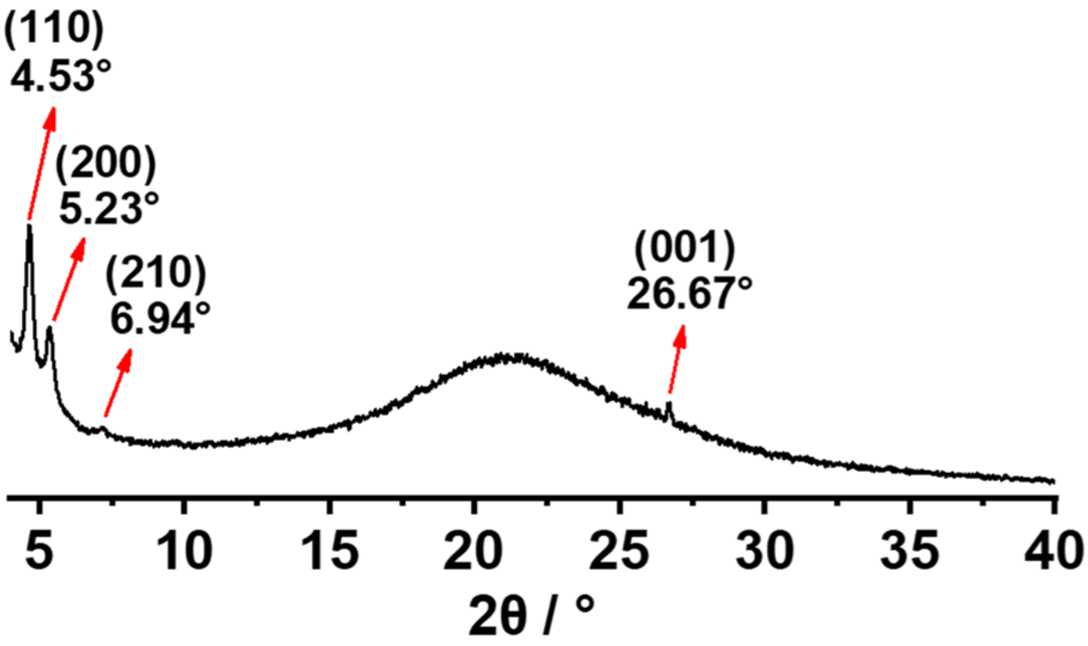

Supplementary Figure 14. Expanded PXRD pattern of ***g*-C$_{40}$N$_3$-COF** from 2θ = 4°~40°.

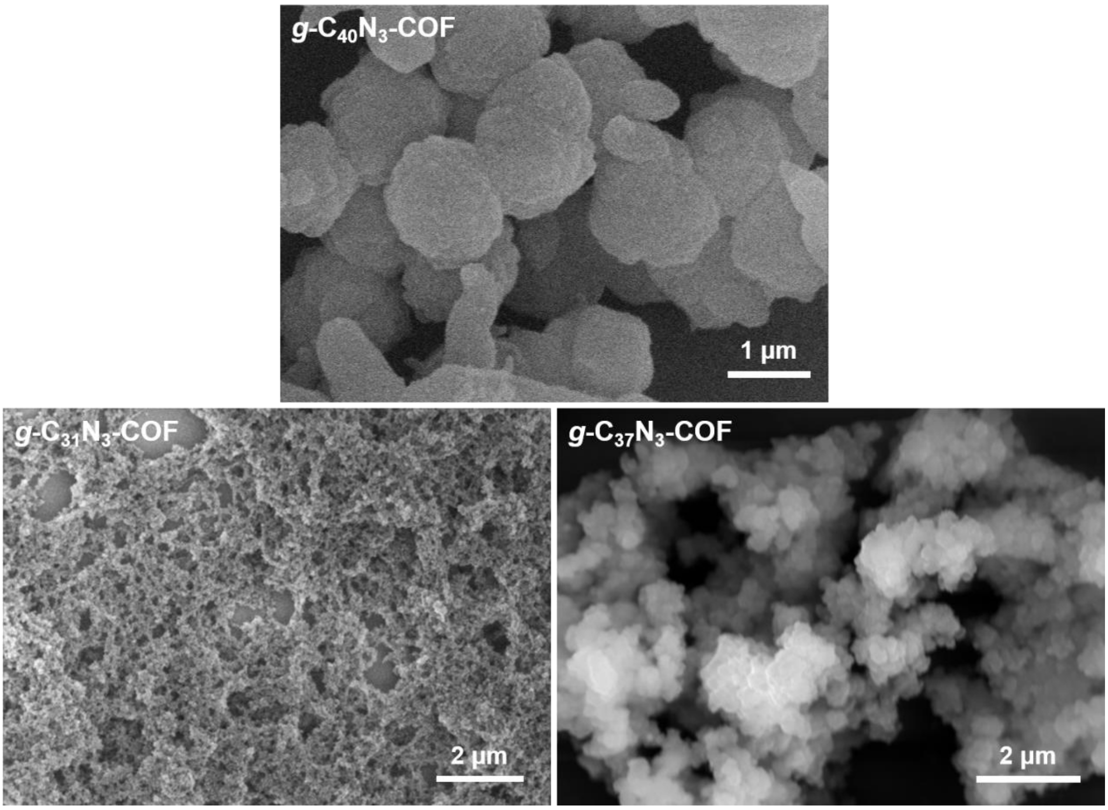

Supplementary Figure 15. SEM images of ***g*-$C_{40}N_3$-COF**, ***g*-$C_{31}N_3$-COF** and ***g*-$C_{37}N_3$-COF**.

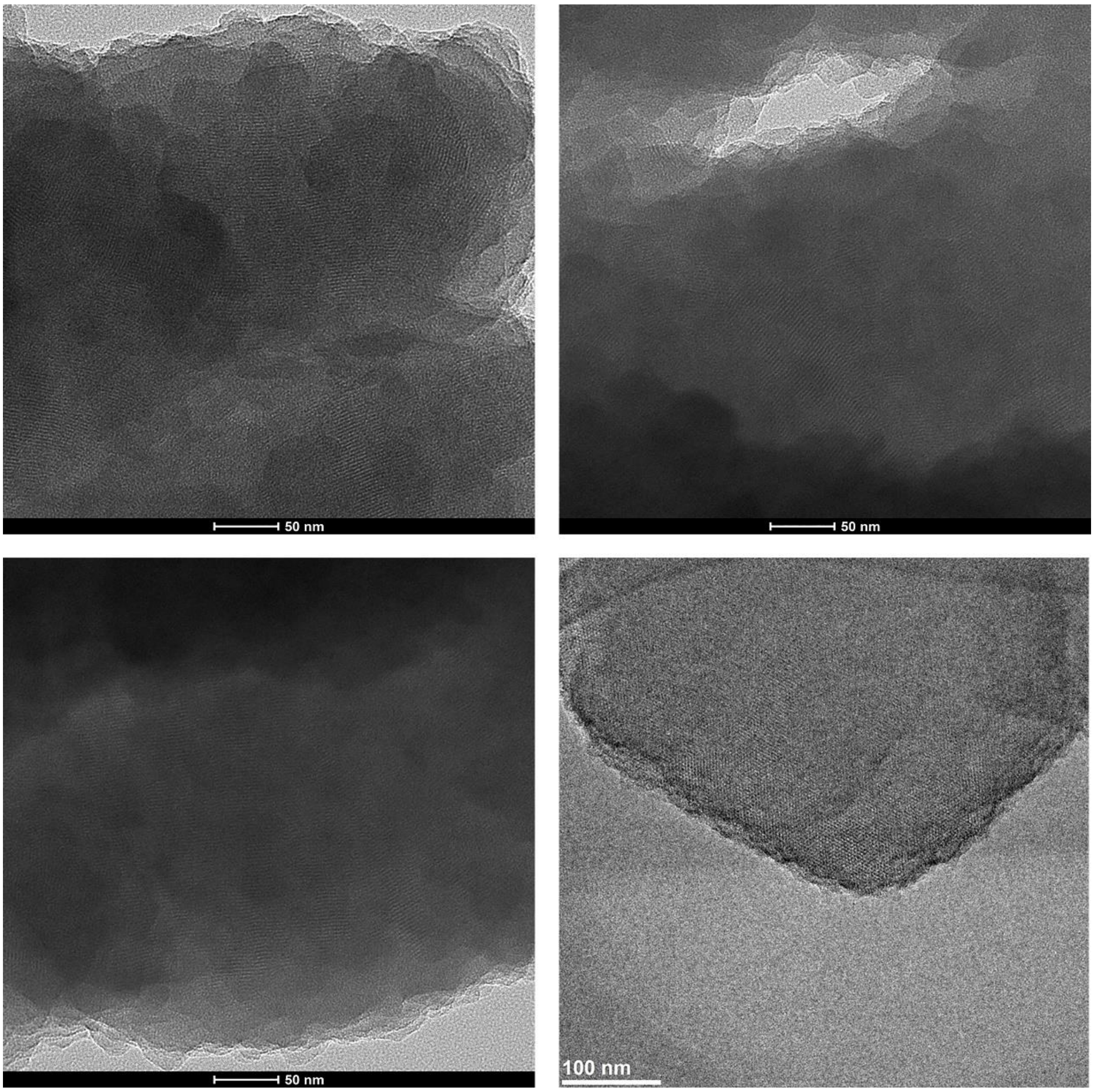

Supplementary Figure 16. TEM images of ***g*-$C_{40}N_3$-COF**.

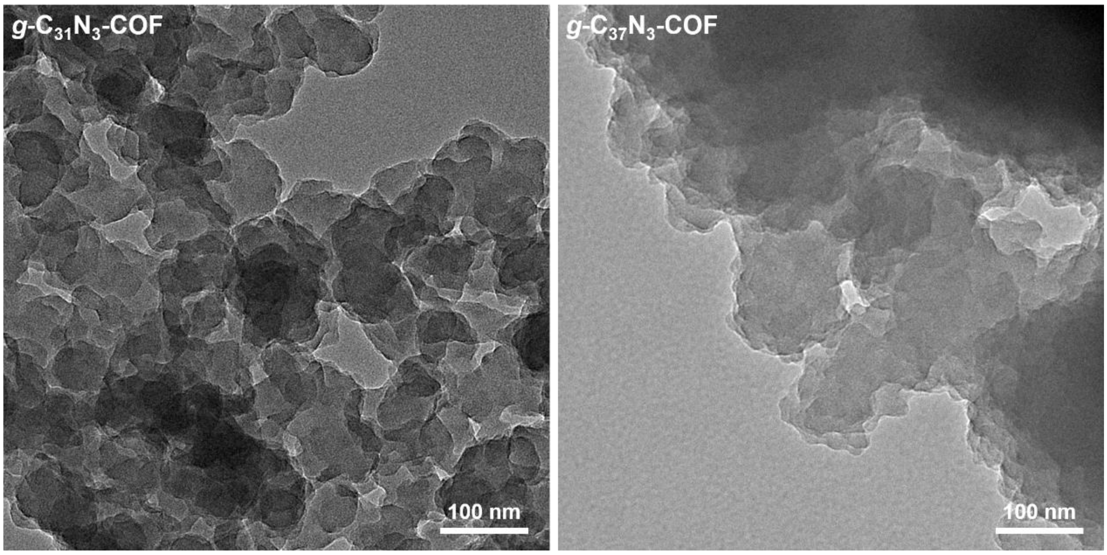

Supplementary Figure 17. TEM images of ***g*-$C_{31}N_3$-COF** and ***g*-$C_{37}N_3$-COF**.

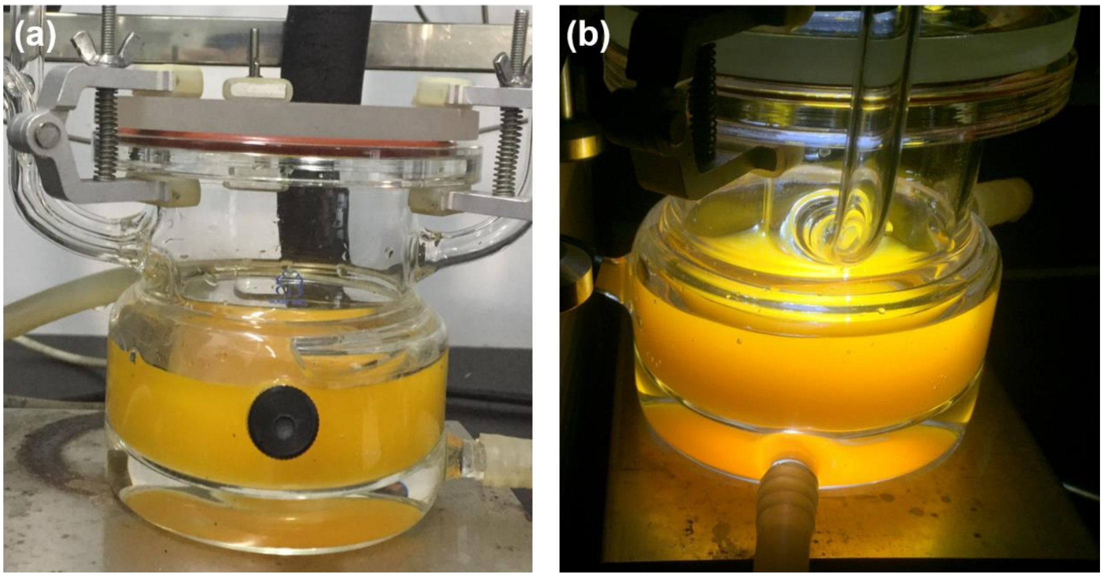

Supplementary Figure 18. The photograph of the reactor setup for hydrogen evolution experiments. (a) under ambient light; (b) irradiation by 300 W Xenon light. The photos show excellent dispersibility of ***g*-C$_{40}$N$_3$-COF** in water/TEoA mixture.

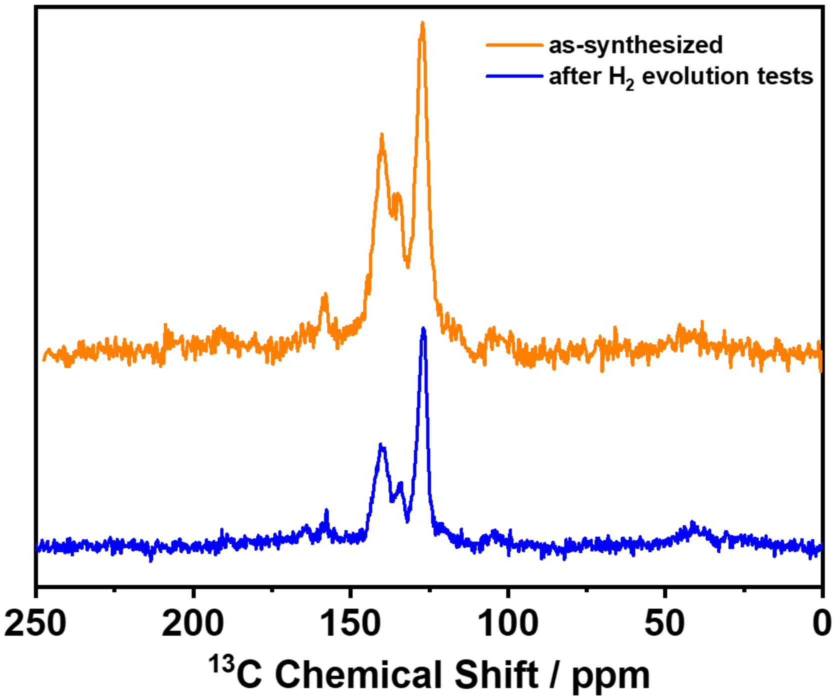

Supplementary Figure 19. ^{13}C CP/MAS solid-state NMR (ssNMR) spectra of ***g*-C$_{40}$N$_3$-COF** as-synthesized (orange) and after long-term photocatalytic hydrogen evolution tests (blue).

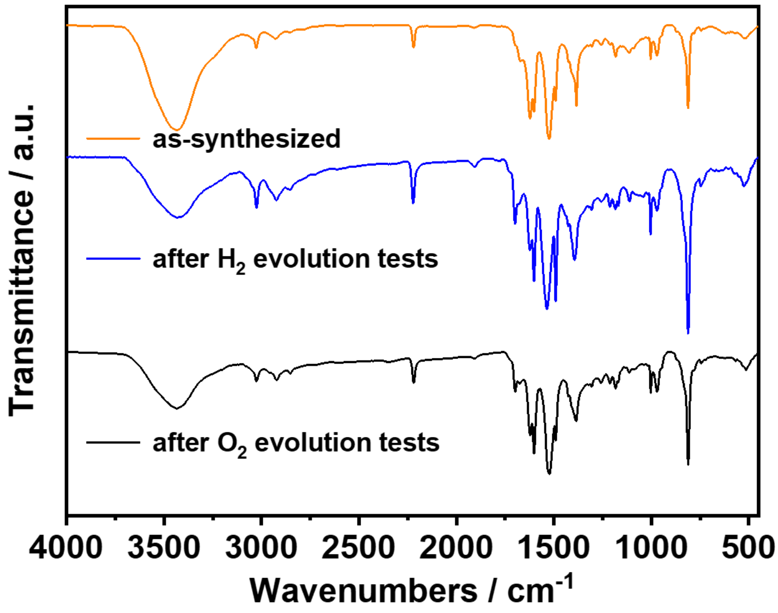

Supplementary Figure 20. FT-IR spectra of ***g*-C$_{40}$N$_3$-COF** as-synthesized (orange), after long-term photocatalytic hydrogen evolution tests (blue) and after long-term photocatalytic oxygen evolution tests (black).

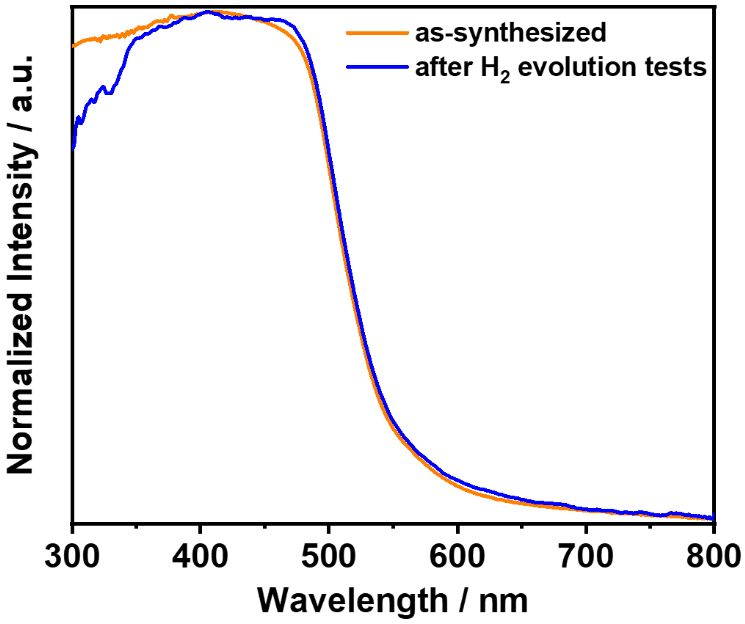

Supplementary Figure 21. UV/vis diffuse reflectance spectra (UV/vis DRS) of ***g*-C$_{40}$N$_3$-COF** as-synthesized (orange) and after long-term photocatalytic hydrogen evolution tests (blue).

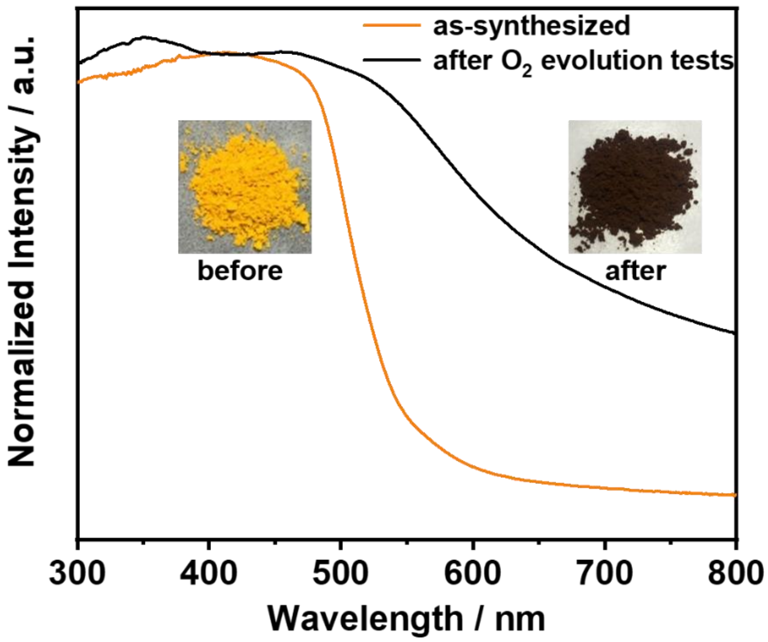

Supplementary Figure 22. UV/vis diffuse reflectance spectra (UV/vis DRS) of ***g*-C$_{40}$N$_3$-COF** as-synthesized (orange) and after long-term photocatalytic oxygen evolution tests (black).

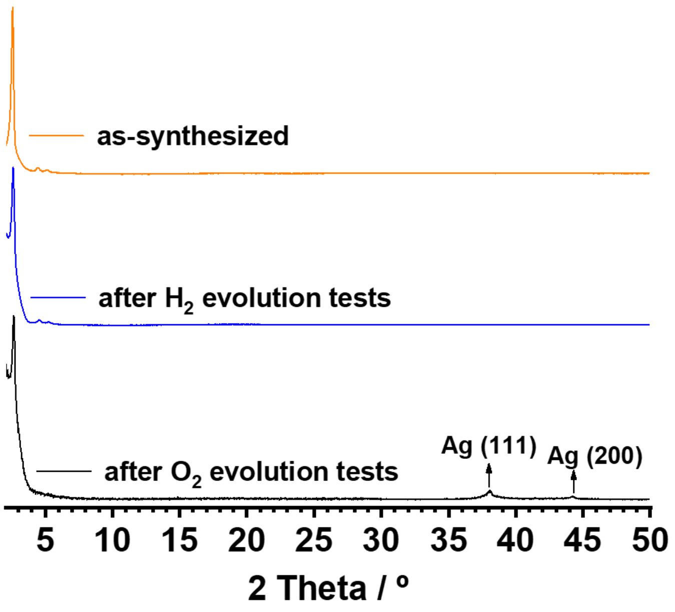

Supplementary Figure 23. PXRD of ***g*-C$_{40}$N$_3$-COF** as-synthesized (orange), after long-term photocatalytic hydrogen evolution tests (blue) and after long-term photocatalytic oxygen evolution tests (black).

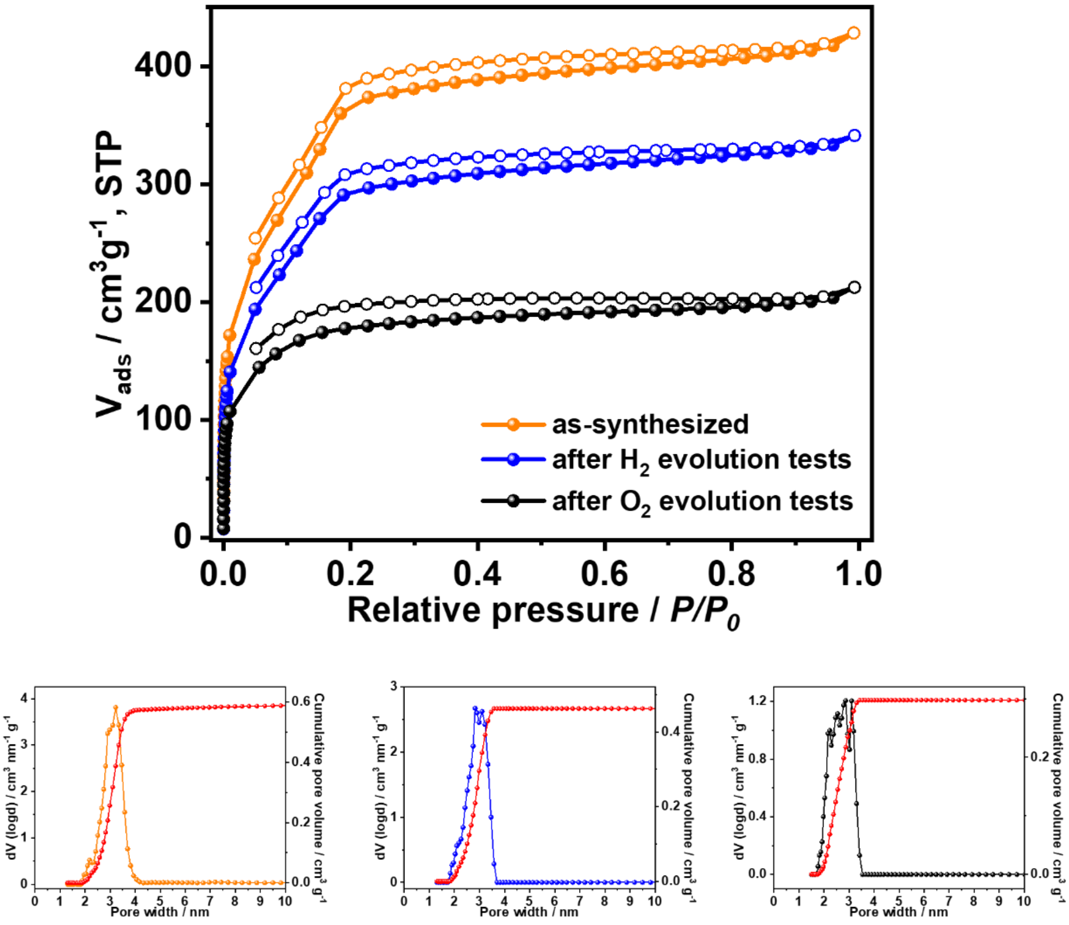

Supplementary Figure 24. Nitrogen adsorption−desorption isotherms and corresponding non-local density functional theory (NLDFT) pore size distributions (PSD) of ***g*-$C_{40}N_3$-COF** as-synthesized (orange), after long-term photocatalytic hydrogen evolution tests (blue) and after long-term photocatalytic oxygen evolution tests (black).

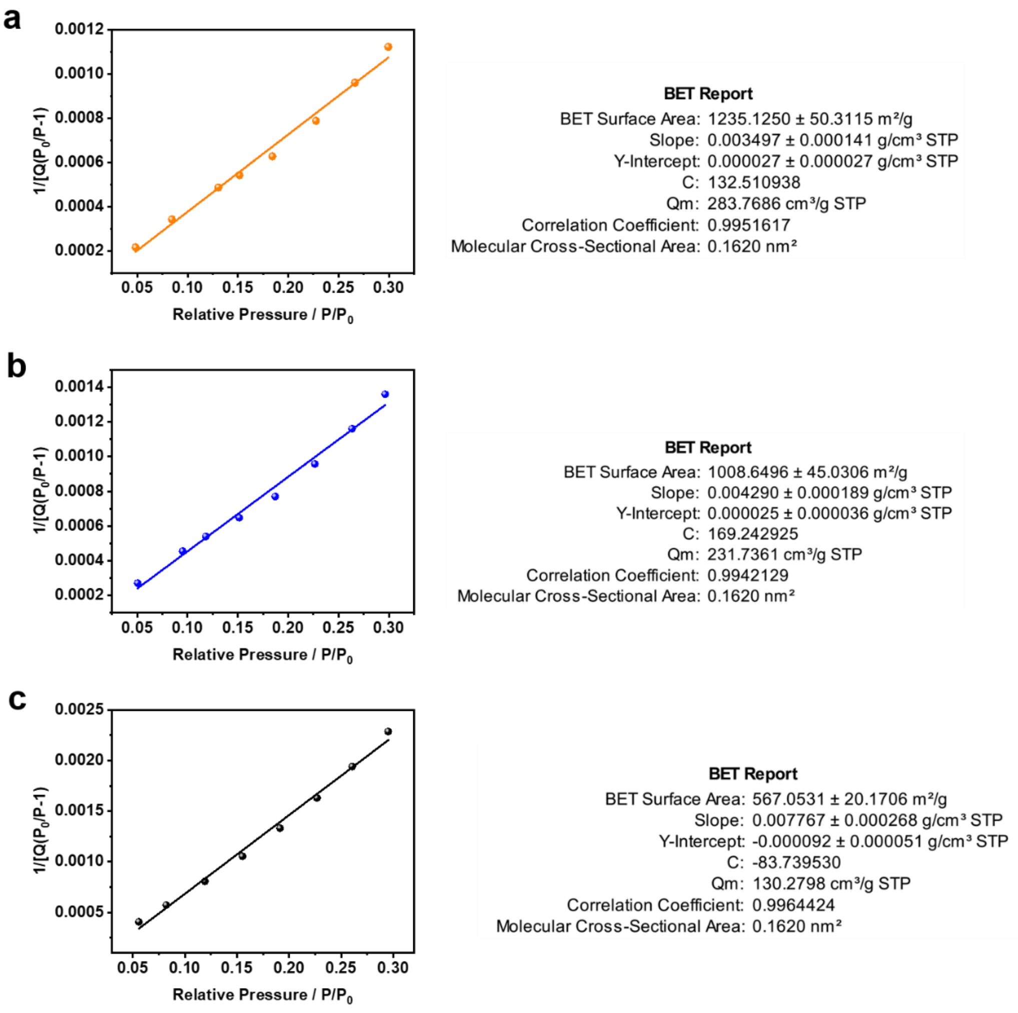

Supplementary Figure 25. Plots of the linear region for the BET equation of ***g*-$C_{40}N_3$-COF** as-synthesized (orange), after long-term photocatalytic hydrogen evolution tests (blue) and after long-term photocatalytic oxygen evolution tests (black).

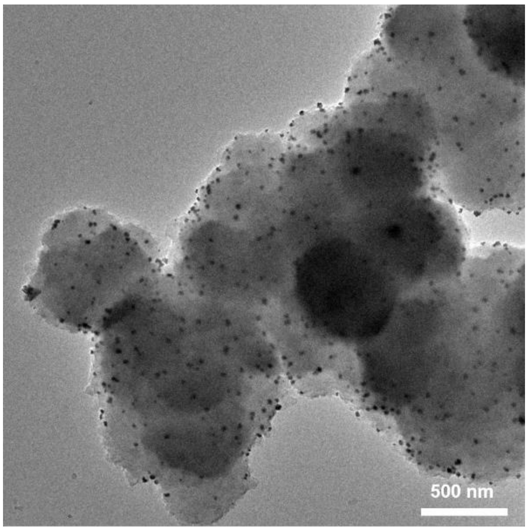

Supplementary Figure 26. TEM image of ***g*-$C_{40}N_3$-COF** after loading with 3 wt% Pt by *in situ* photodeposition method using H_2PtCl_6, showing uniform dispersion of Pt nanoparticles.

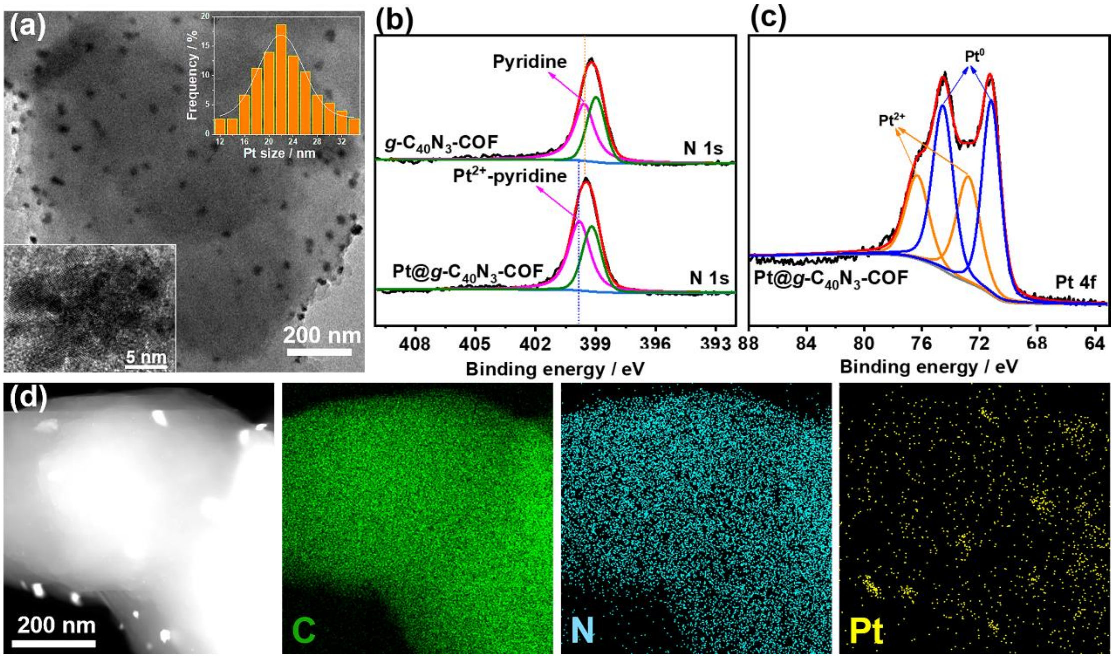

Supplementary Figure 27. (a) TEM image of **Pt@*g*-$C_{40}N_3$-COF**. Left inset shows the magnified image of a Pt particle, showing its dendritic structure. Right inset shows the Pt particle size distribution. (b) Comparison of XPS spectra of N 1*s* orbitals of ***g*-$C_{40}N_3$-COF** and **Pt@*g*-$C_{40}N_3$-COF**. (c) Pt 4*f* core level XPS spectra of **Pt@ *g*-$C_{40}N_3$-COF**. (d) high angle annular dark field (HAADF) TEM image and elemental mapping of **Pt@*g*-$C_{40}N_3$-COF.**

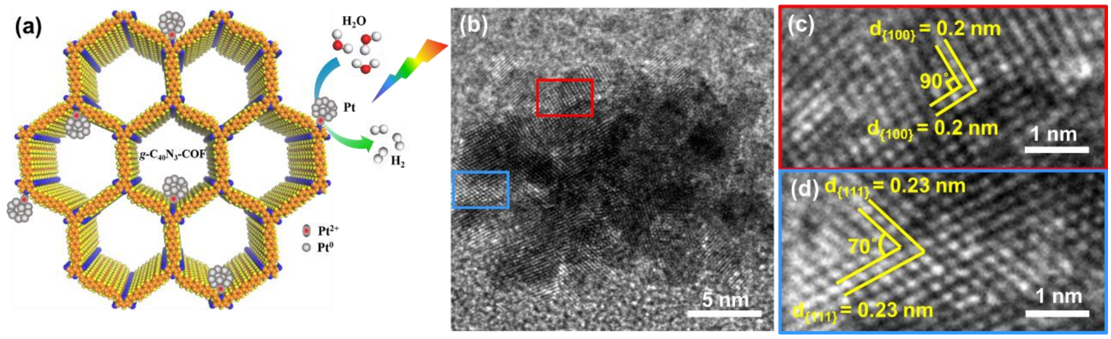

Supplementary Figure 28. (a) Schematic illustration of **Pt@*g*-$C_{40}N_3$-COF** for hydrogen evolution from water under visible light irradiation. (b) HRTEM image of Pt nanoparticle. (c) Enlarged HRTEM image of the red square area in (b), showing the {100} planes of Pt with d spacing of 0.2 nm. (d) Enlarged HRTEM image of the blue square area in (b), showing the {111} planes of Pt with d spacing of 0.23 nm.

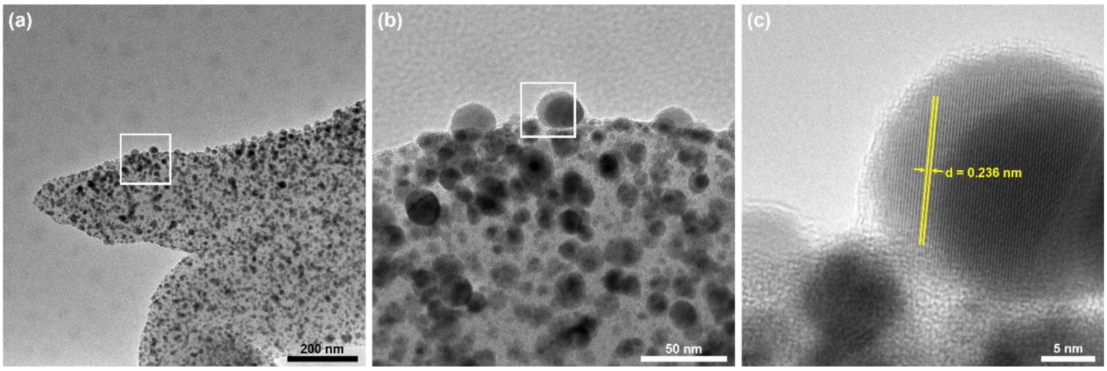

Supplementary Figure 29. (a) TEM image of ***g*-C$_{40}$N$_3$-COF** after long-term photocatalytic oxygen evolution tests. The dense black spots indicate Ag nanoparticles. (b) Enlarged TEM image of white square area in (a), showing Ag spheric nanoparticles deposited on the surface of COF catalyst. (c) Enlarged TEM image of white square area in (b). The lattice fringes correspond to Ag(111) facet.

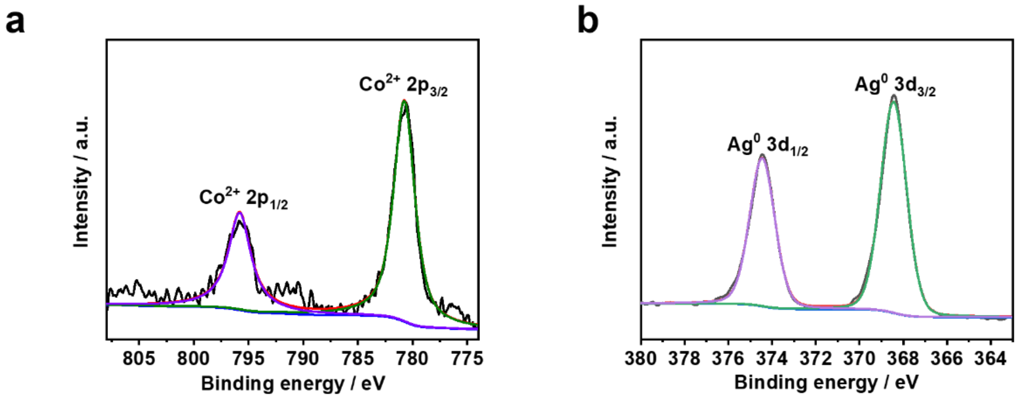

Supplementary Figure 30. (a) Co 2*p* XPS spectra of ***g*-C$_{40}$N$_3$-COF** after long-term photocatalytic oxygen evolution tests. (b) Ag 3*d* XPS spectra of ***g*-C$_{40}$N$_3$-COF** after long-term photocatalytic oxygen evolution tests.

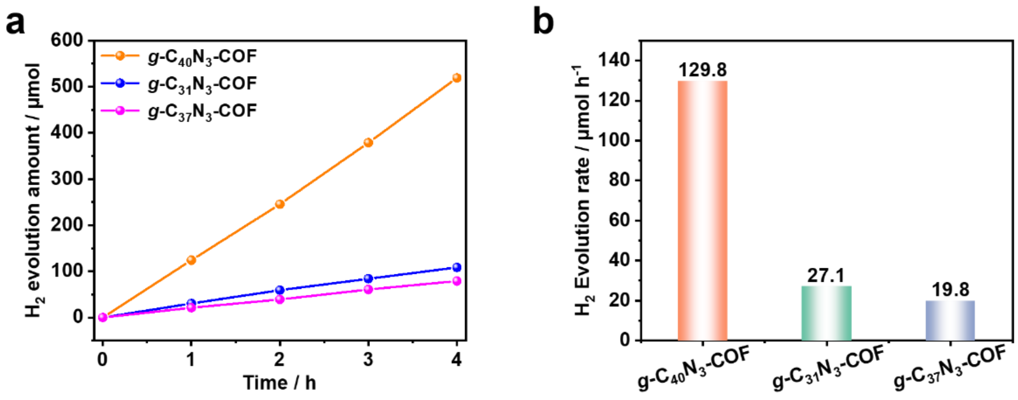

Supplementary Figure 31. (a) 4-h H_2 evolution arrays using 50 mg of 3 wt% Pt modified ***g*-C$_{40}$N$_3$-COF**, ***g*-C$_{31}$N$_3$-COF** and ***g*-C$_{37}$N$_3$-COF**. (b) Corresponding H_2 evolution rates.

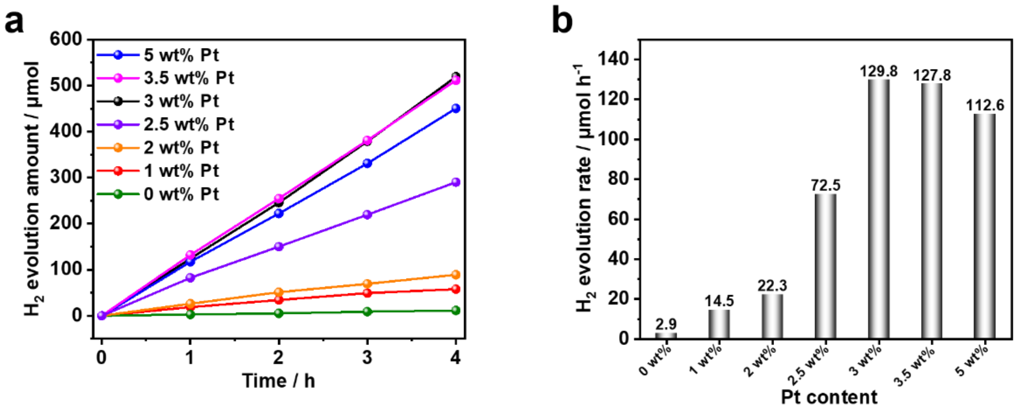

Supplementary Figure 32. (a) The first four-hour hydrogen evolution arrays from 100 mL H_2O and 10 mL TEoA mixture using 50 mg x wt% Pt modified ***g*-C$_{40}$N$_3$-COF** (x = 0, 1, 2, 2.5, 3, 3.5, 5). (b) The average hydrogen evolution rate of x wt% Pt modified ***g*-C$_{40}$N$_3$-COF** (x = 0, 1, 2, 2.5, 3, 3.5, 5), indicating the optimal Pt loading amount is 3 wt%.

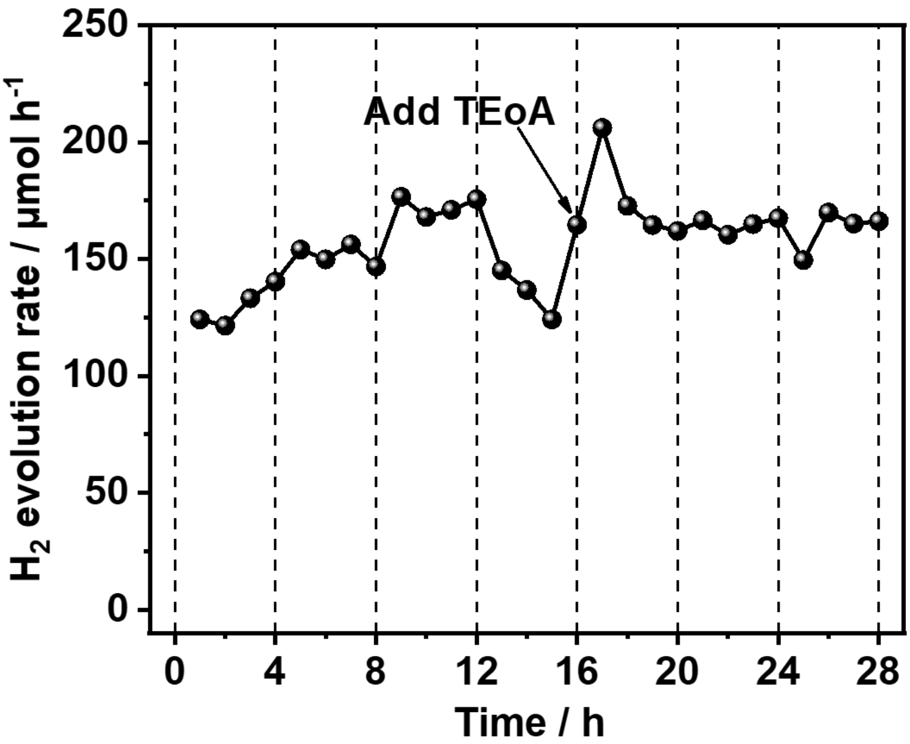

Supplementary Figure 33. The amount of hourly evolved hydrogen from 100 mL H_2O and 10 mL TEoA mixture using 50 mg 3 wt% Pt modified ***g*-C$_{40}$N$_3$-COF** during the long-term photocatalytic test.

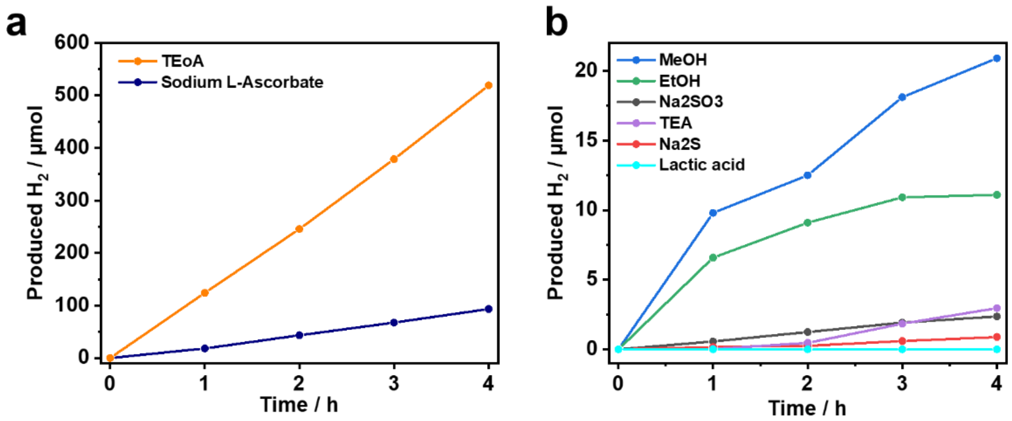

Supplementary Figure 34. Hydrogen evolution arrays using 50 mg of 3 wt% Pt modified ***g*-C$_{40}$N$_3$-COF** with different sacrificial agents.

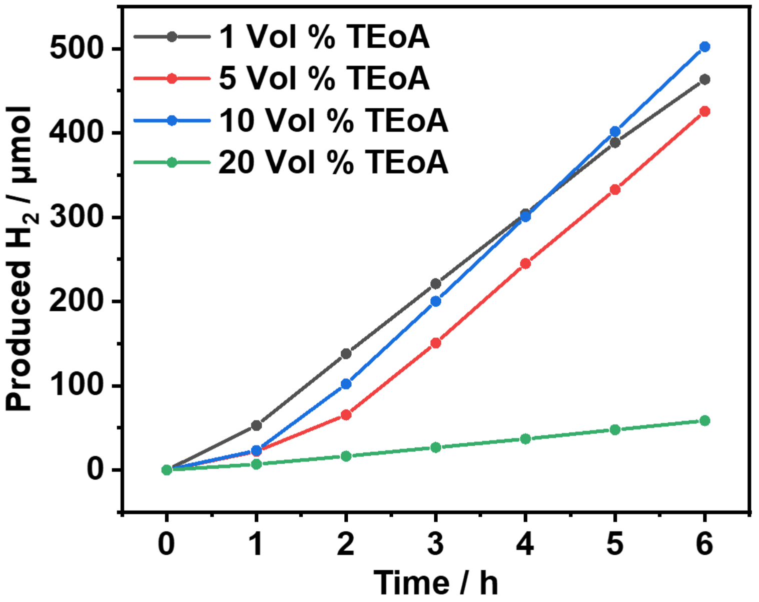

Supplementary Figure 35. The first six-hour hydrogen evolution arrays from 110 mL of an aqueous solution of TEoA with different volume concentrations (x Vol %, x = 1, 5, 10, 20) using 50 mg ***g*-C$_{40}$N$_3$-COF**, 3 wt% Pt was *in situ* photodeposition onto the surface of catalyst by adding hexachloroplatinic acid aqueous solution.

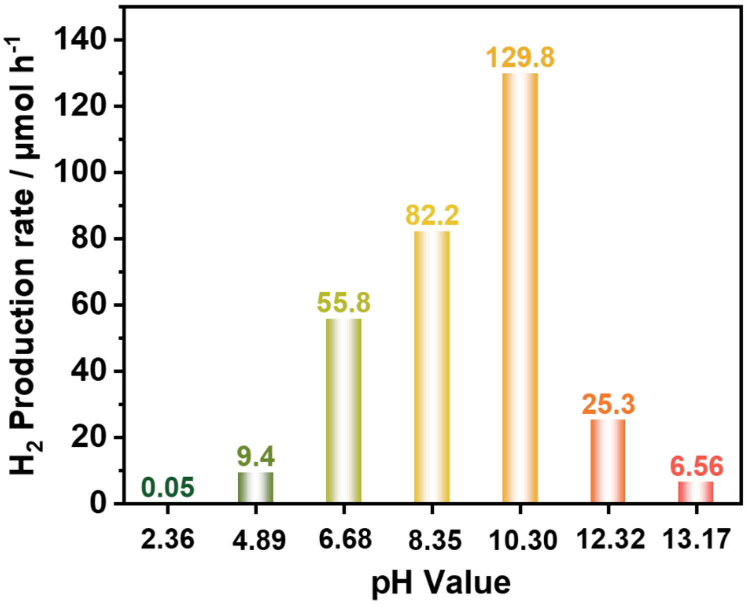

Supplementary Figure 36. Average hydrogen evolution rate for 50 mg 3 wt% Pt modified ***g*-C$_{40}$N$_3$-COF** at different pH values under a 300-W Xe lamp irradiation with cut-off filter $\lambda > 420$ nm using 10 Vol% TEoA as sacrificial electron donor. The pH value of unadjusted 10 Vol% TEoA aqueous solution is 10.30. The pH values were adjusted by 2 M aqueous solution of NaOH or H_2SO_4 and measured by pH meter.

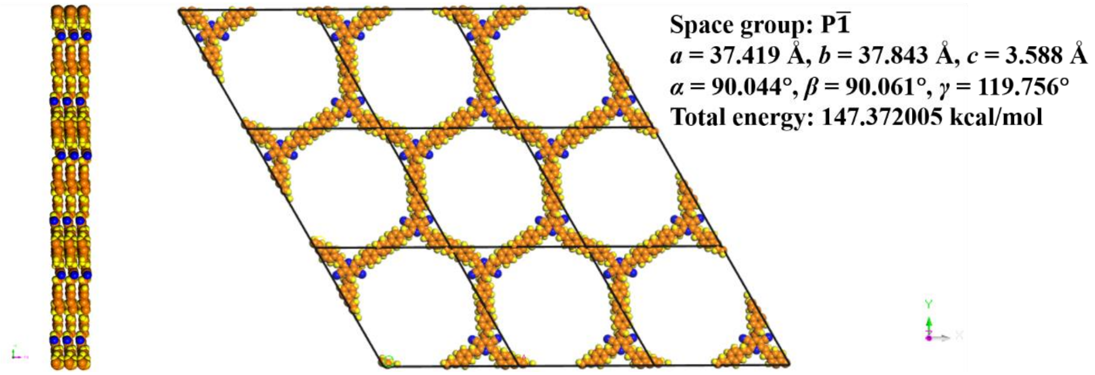

Supplementary Figure 37. The view of eclipsed AA-stacking model of ***g*-C$_{40}$N$_3$-COF** with $P\bar{1}$ space group.

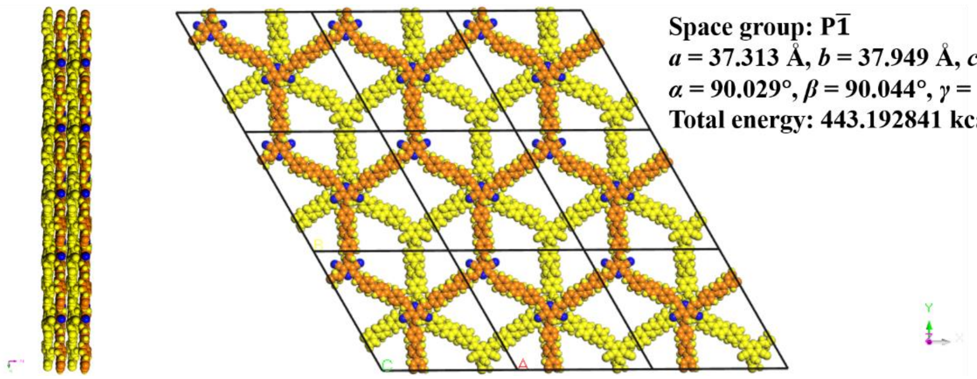

Supplementary Figure 38. The view of staggered AB-stacking model of ***g*-C$_{40}$N$_3$-COF** with $P\bar{1}$ space group.

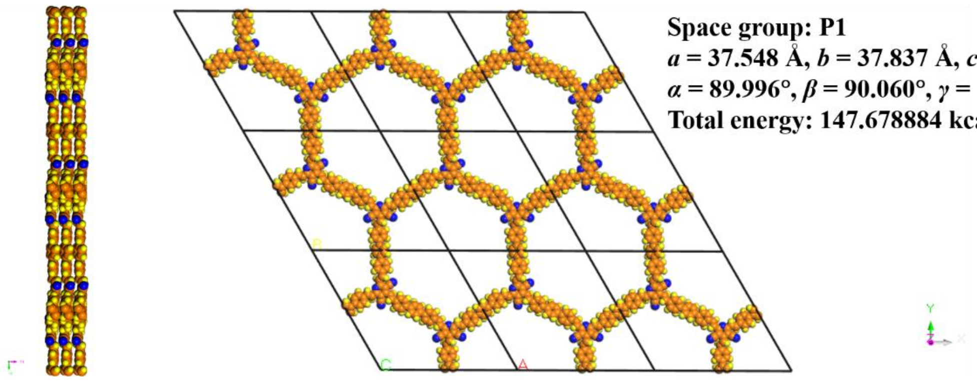

Supplementary Figure 39. The view of eclipsed AA-stacking model of ***g*-C$_{40}$N$_3$-COF** with $P1$ space group.

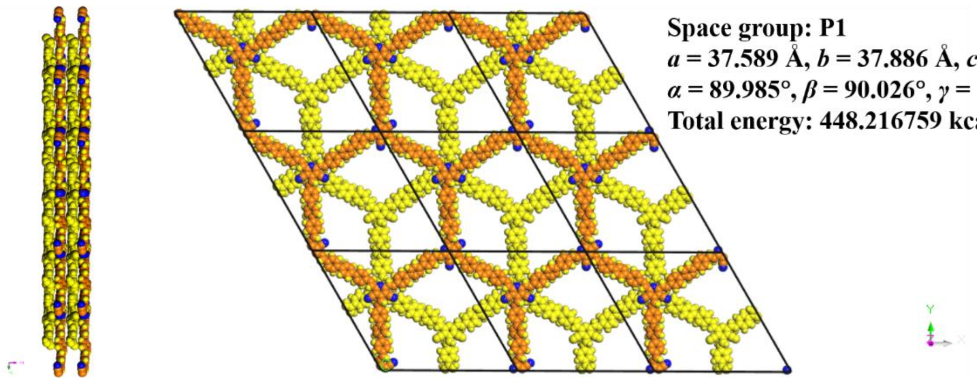

Supplementary Figure 40. The view of staggered AB-stacking model of ***g*-C$_{40}$N$_3$-COF** with $P1$ space group.

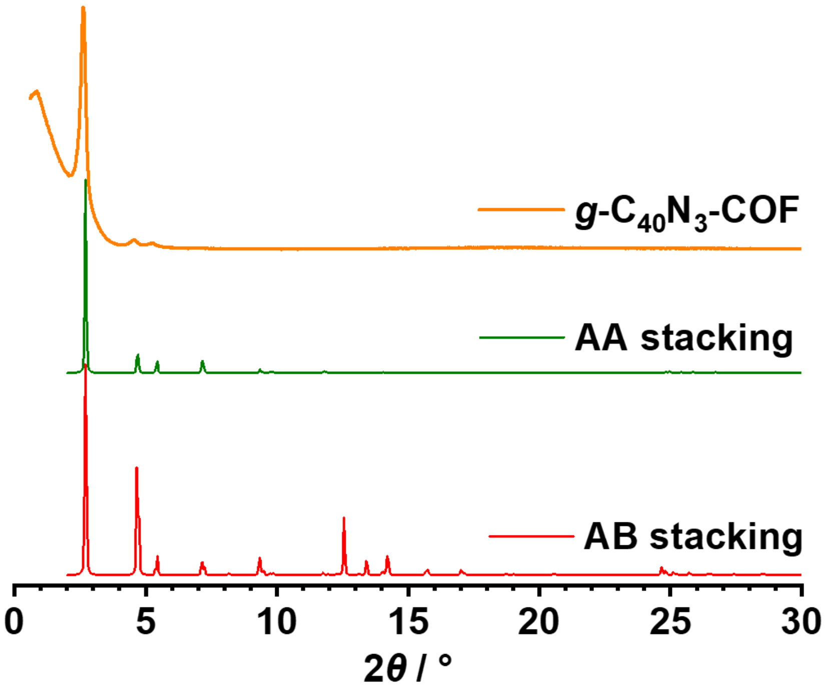

Supplementary Figure 41. Simulated X-ray diffraction patterns for ***g*-C$_{40}$N$_3$-COF** with $P\bar{1}$ space group adopting fully eclipsed AA-stacking (green) and staggered AB-stacking (red) arrangement compared to the experimentally obtained pattern (orange).

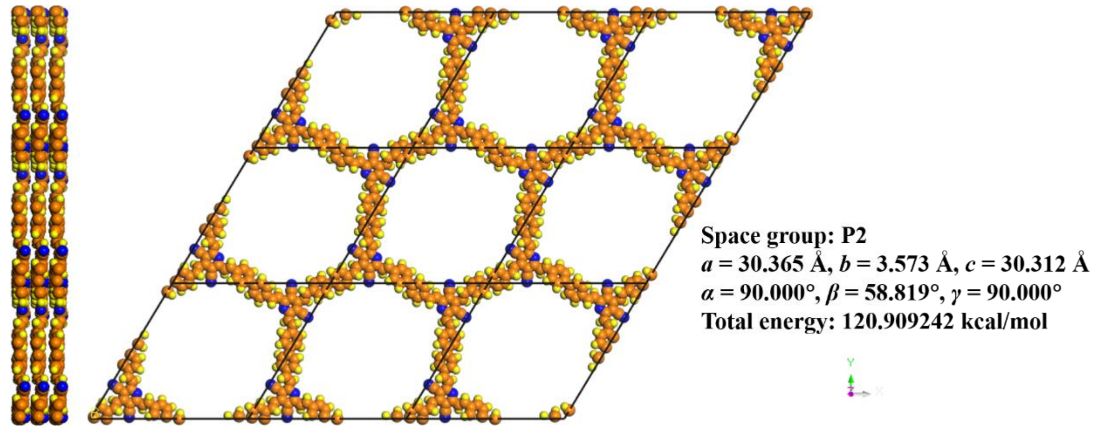

Supplementary Figure 42. The view of eclipsed AA-stacking model of ***g*-$C_{31}N_3$-COF** with *P*2 space group.

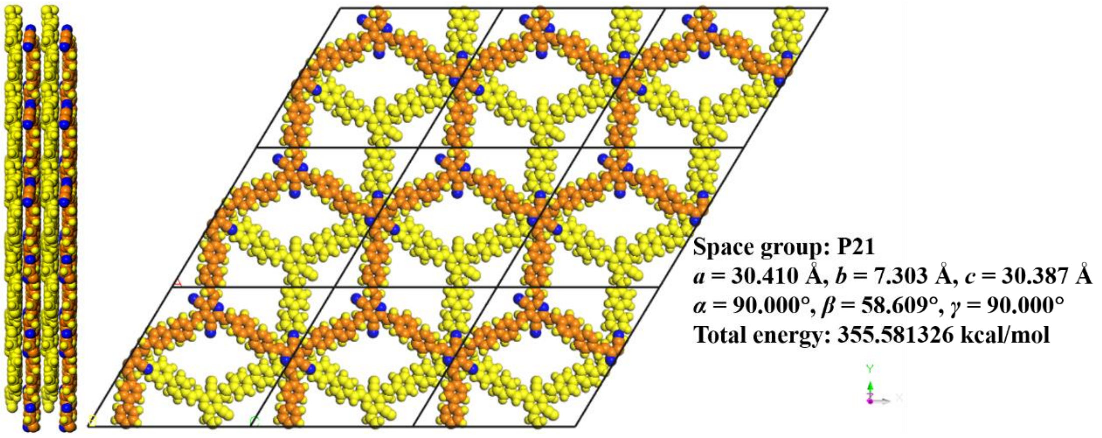

Supplementary Figure 43. The view of staggered AB-stacking model of ***g*-$C_{31}N_3$-COF** with *P*21 space group.

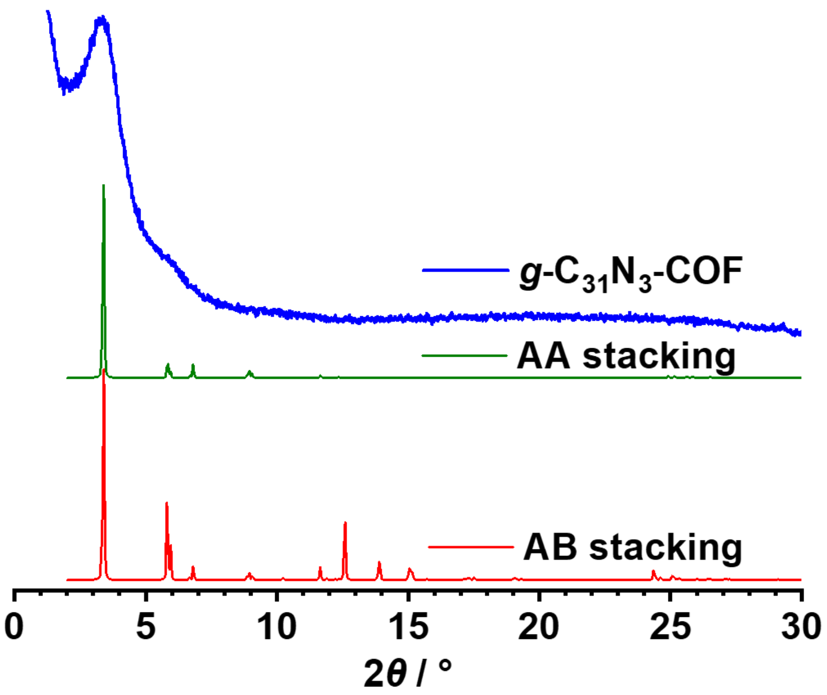

Supplementary Figure 44. Simulated X-ray diffraction patterns for ***g*-$C_{31}N_3$-COF** adopting fully eclipsed AA-stacking (green) and staggered AB-stacking (red) arrangement compared to the experimentally obtained pattern (blue).

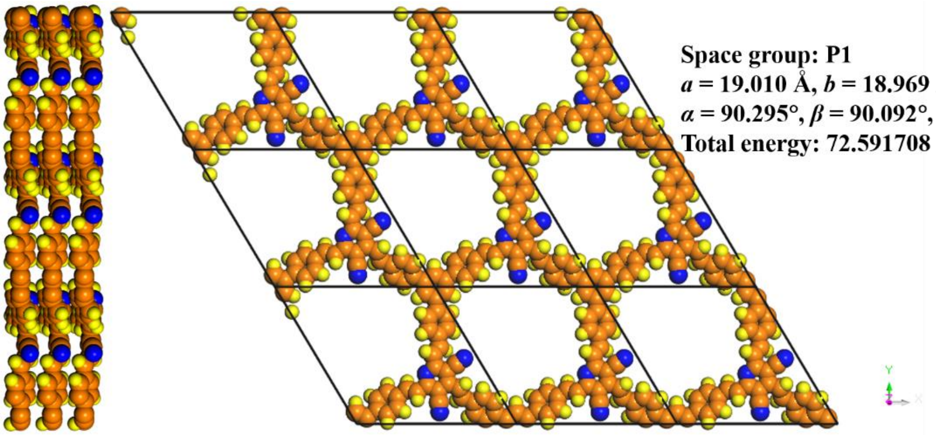

Supplementary Figure 45. The view of eclipsed AA-stacking model of ***g*-$C_{37}N_3$-COF** with *P*1 space group.

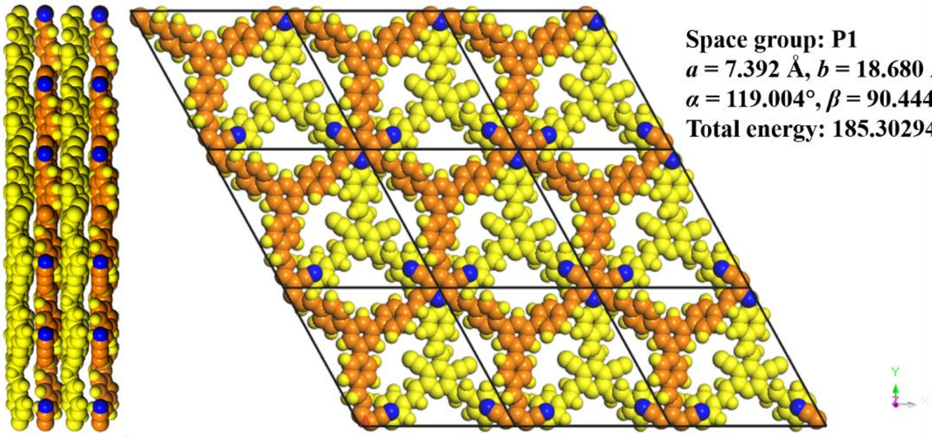

Supplementary Figure 46. The view of staggered AB-stacking model of ***g*-$C_{37}N_3$-COF** with *P*1 space group.

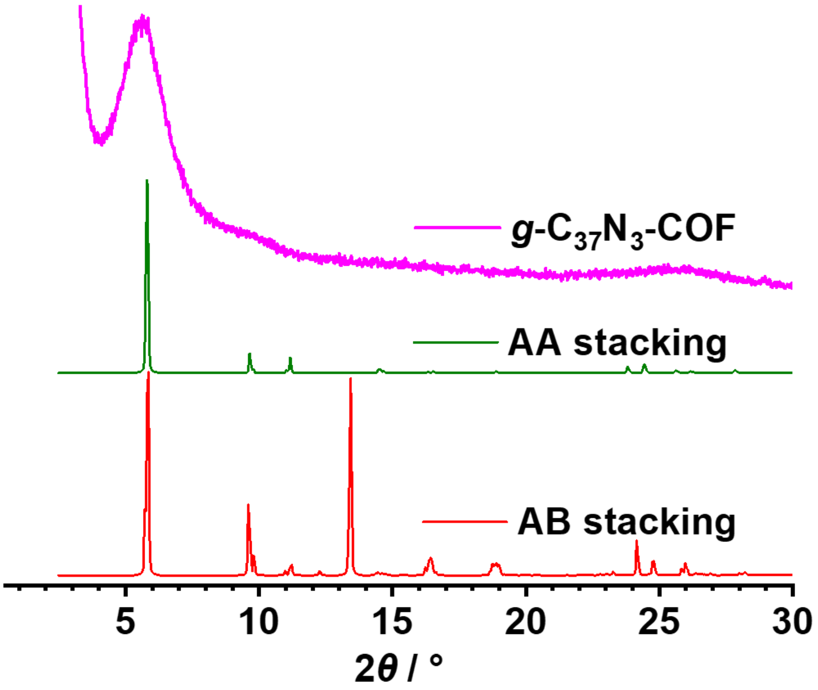

Supplementary Figure 47. Simulated X-ray diffraction patterns for ***g*-$C_{37}N_3$-COF** adopting fully eclipsed AA-stacking (green) and staggered AB-stacking (red) arrangement compared to the experimentally obtained pattern (purple).

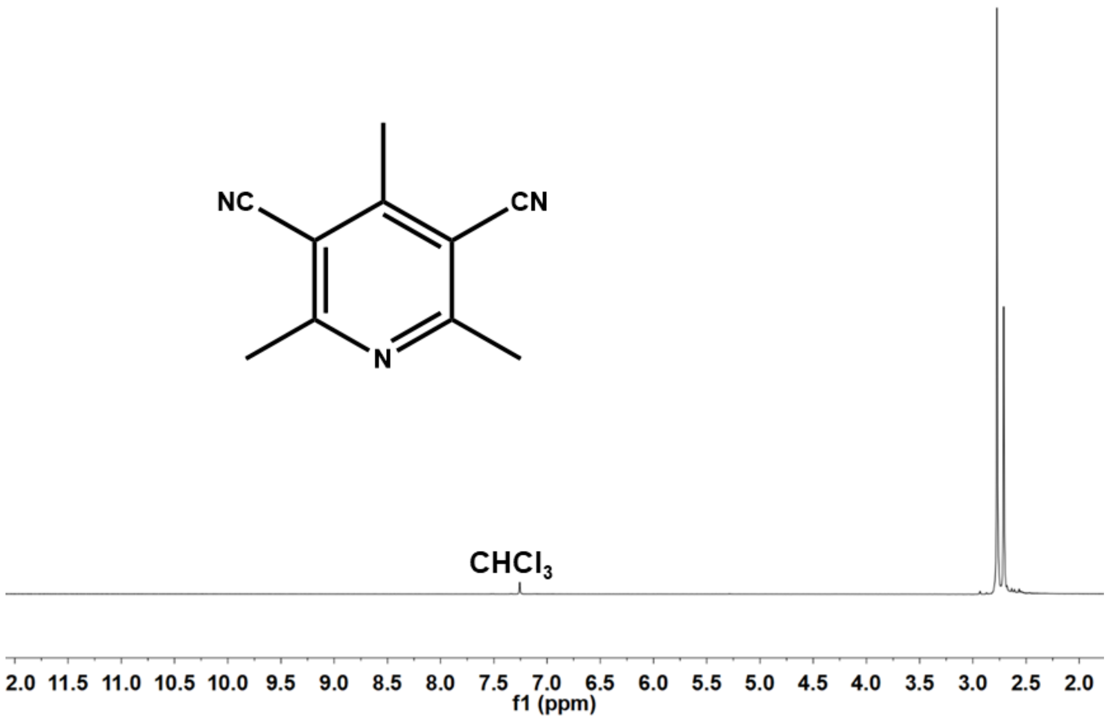

Supplementary Figure 48. ^{1}H NMR spectra of 3,5-dicyano-2,4,6-trimethylpyridine (**DCTMP**) in $CDCl_3$.

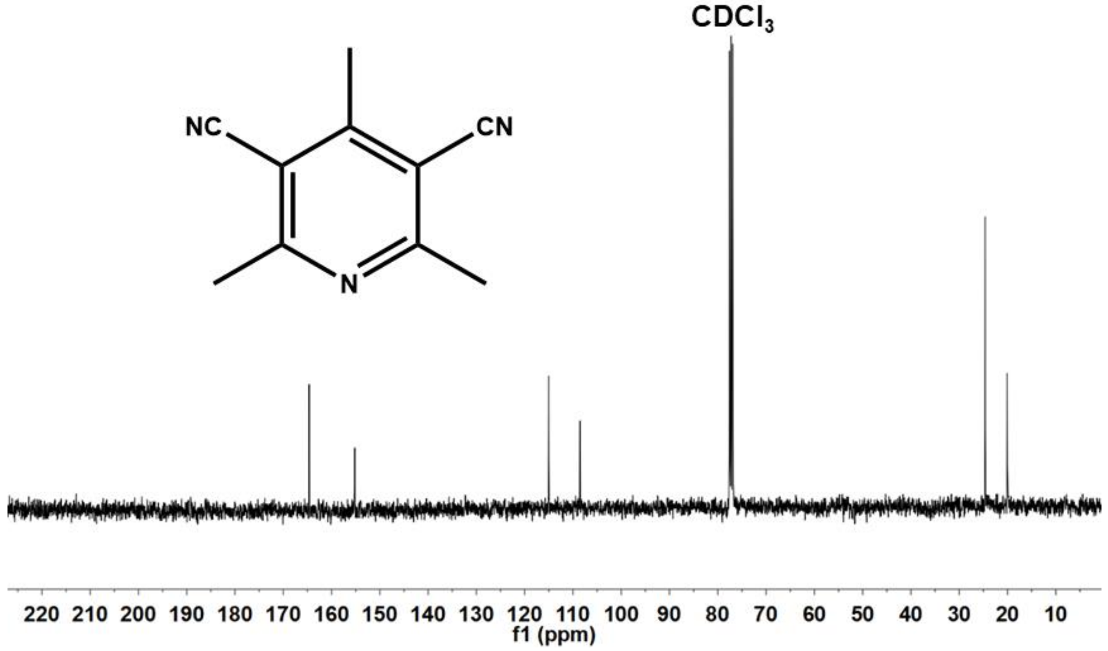

Supplementary Figure 49. ^{13}C NMR spectra of 3,5-dicyano-2,4,6-trimethylpyridine (**DCTMP**) in $CDCl_3$.

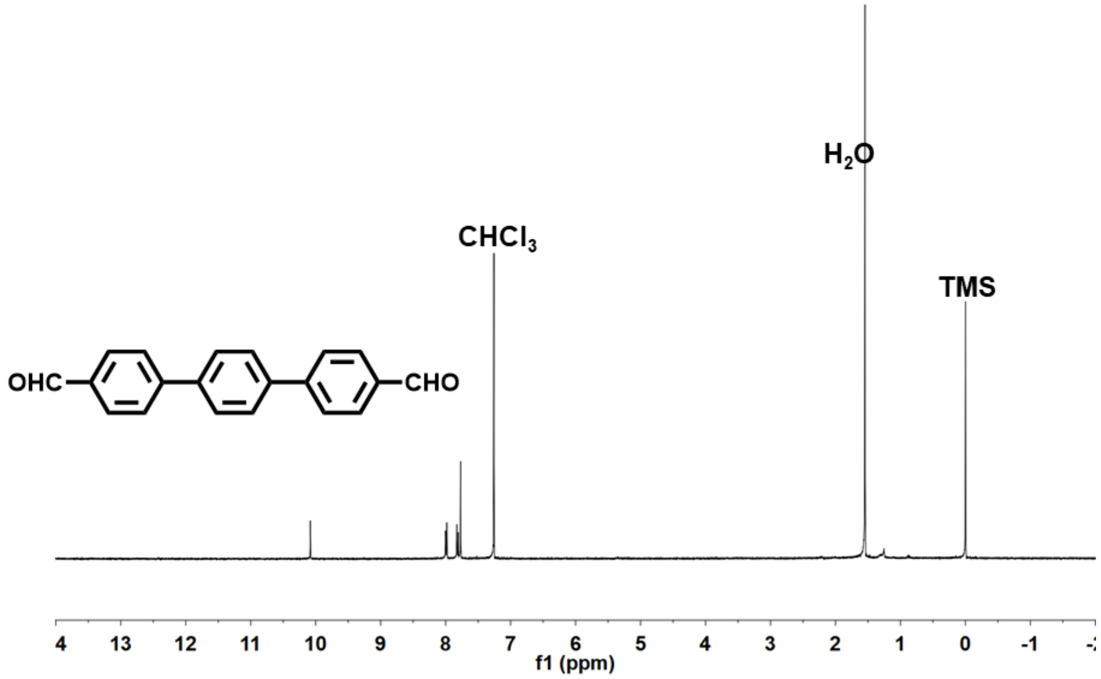

Supplementary Figure 50. ^{1}H NMR spectra of 4,4''-diformyl-*p*-terphenyl (**DFPTP**) in $CDCl_3$.

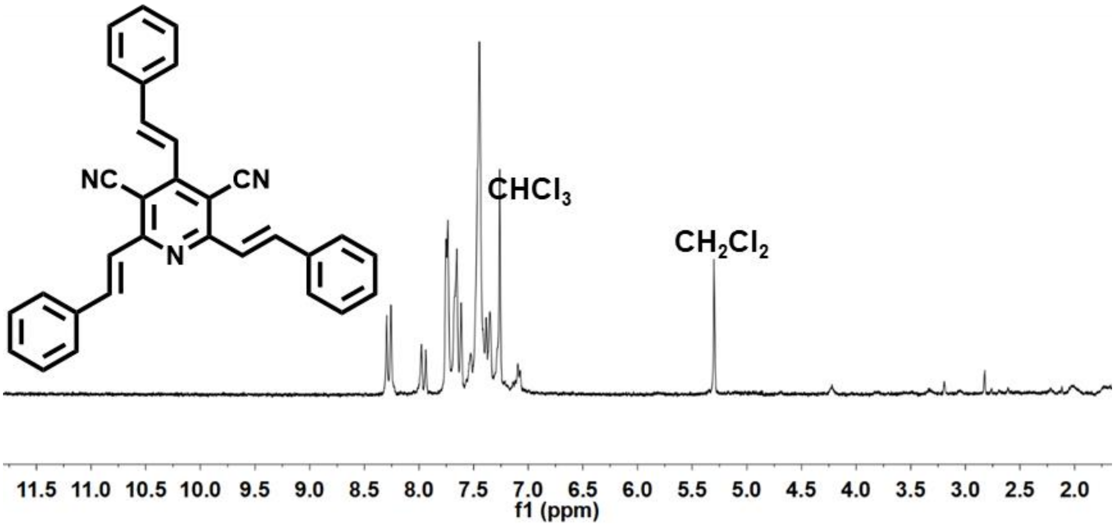

Supplementary Figure 51. ^{1}H NMR spectra of 3,5-dicyano-2,4,6-tristyrylpyridine (model compound) in $CDCl_3$.

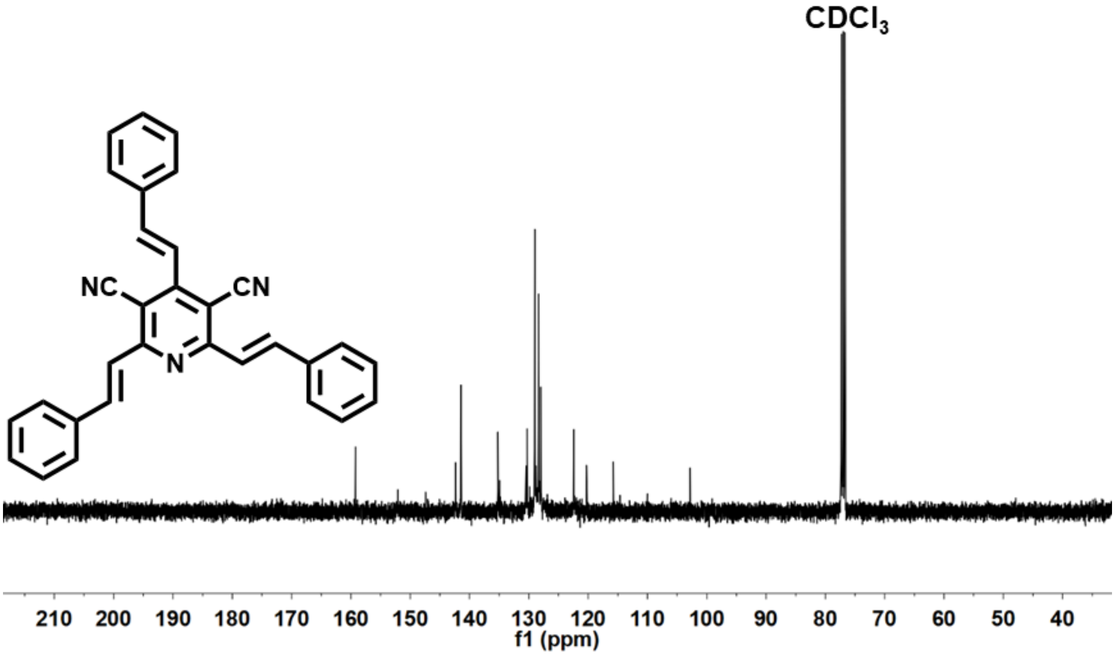

Supplementary Figure 52. ^{13}C NMR spectra of 3,5-dicyano-2,4,6-tristyrylpyridine (model compound) in $CDCl_3$.

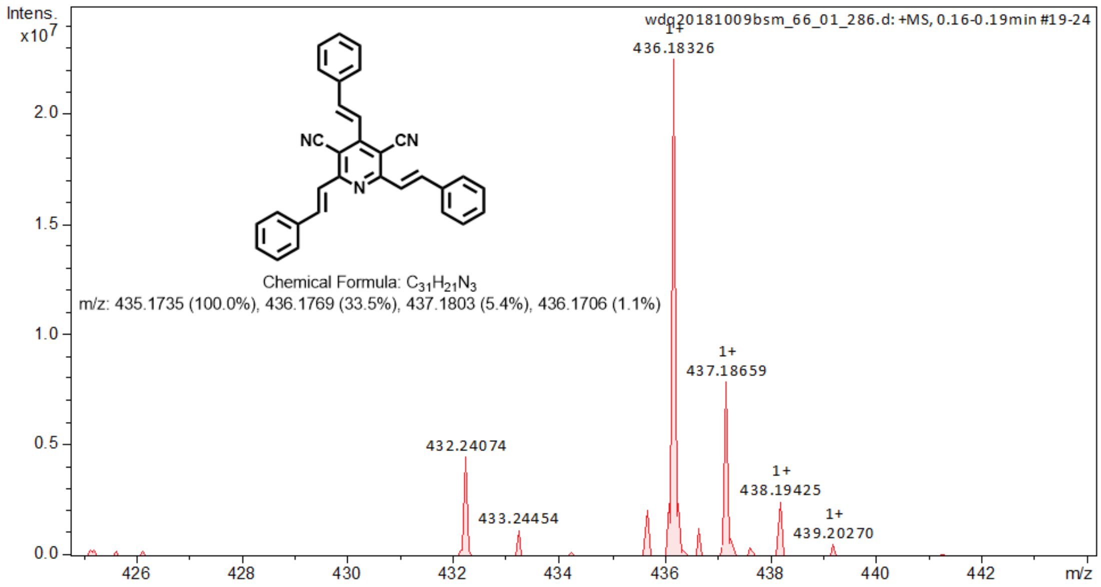

Supplementary Figure 53. HR-MS of 3,5-dicyano-2,4,6-tristyrylpyridine (model compound).

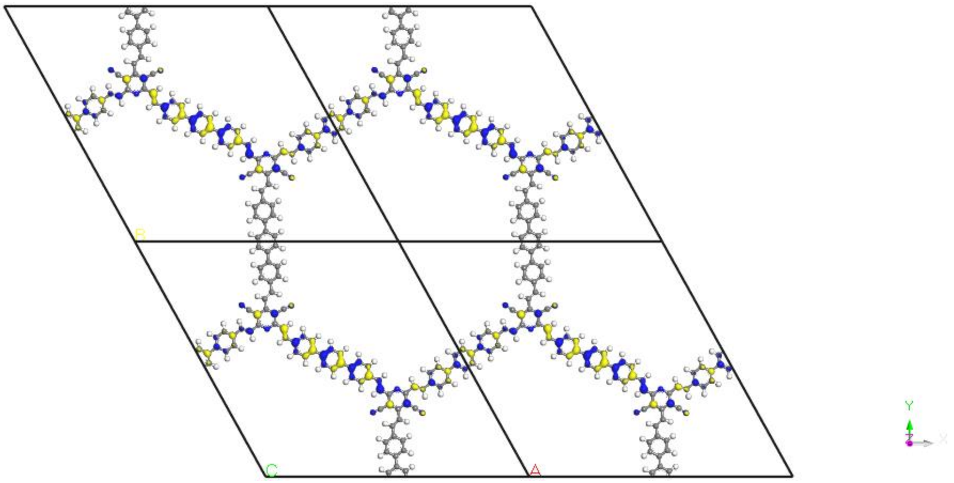

Supplementary Figure 54. Highest occupied molecular orbital (HOMO) of ***g*-C$_{40}$N$_3$-COF**.

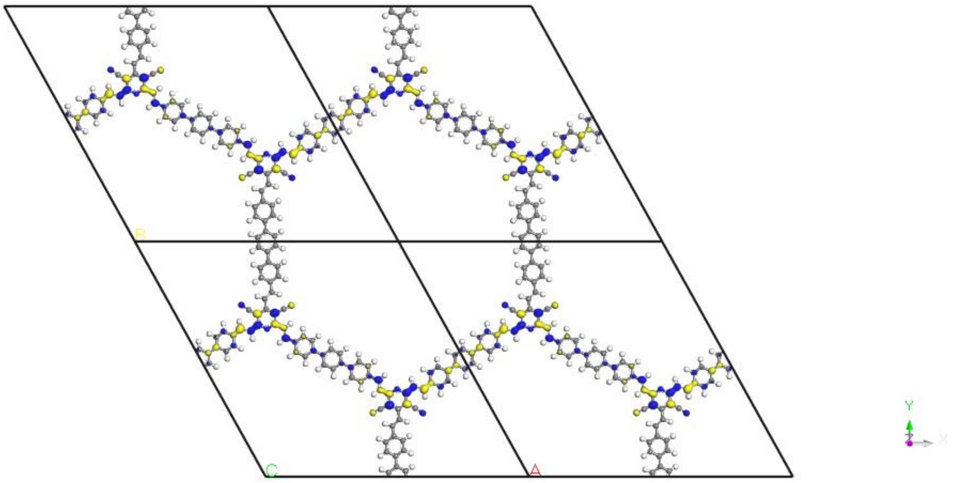

Supplementary Figure 55. Lowest unoccupied molecular orbital (LUMO) of ***g*-C$_{40}$N$_3$-COF**.

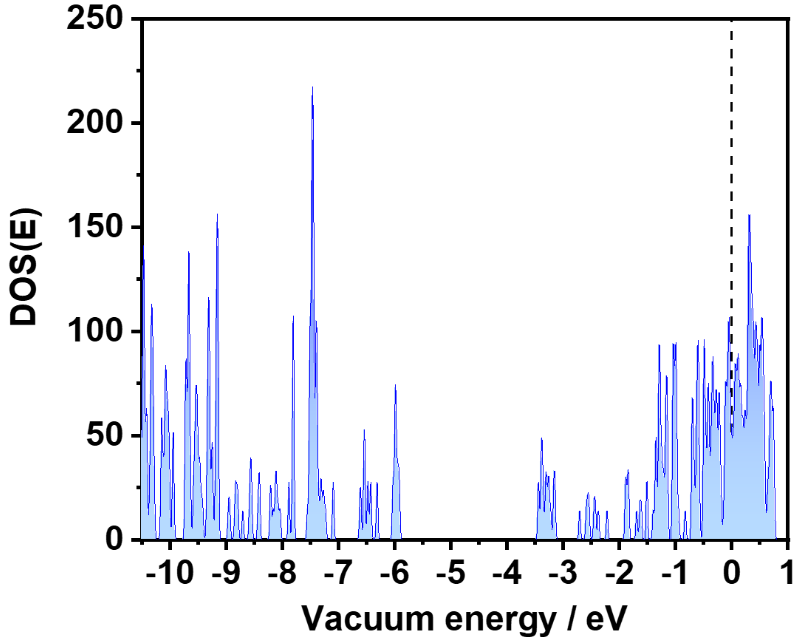

Supplementary Figure 56. Total DOS of ***g*-C$_{40}$N$_3$-COF** with respect to vacuum level predicted by periodic DFT (HSE06) using the Vienna *ab initio* Simulation Package (VASP). The energy levels of VBM and CBM are -5.824 eV for E_{VB} and -3.523 eV for E_{CB}.

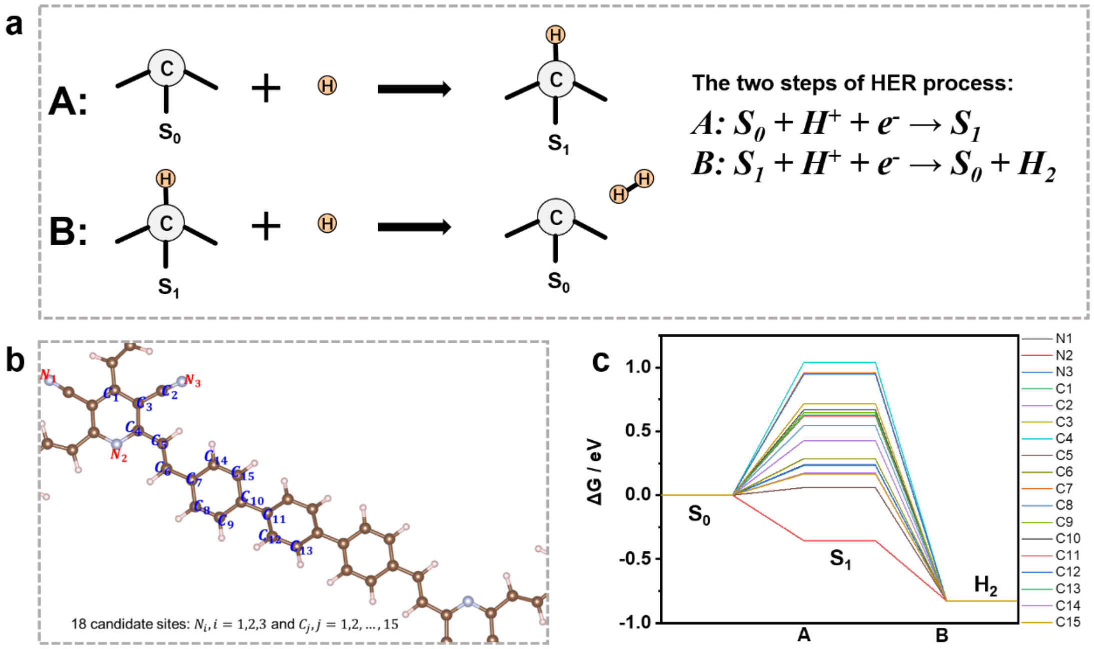

Supplementary Figure 57. (a) Proposed photocatalytic process of hydrogen evolution reaction. $\mathbf{S_0}$ is the bare surface. $\mathbf{S_1}$ is the structure of intermediate state. (b) 18 candidate active sites for H_2 evolution on ***g*-$C_{40}N_3$-COF**. (c) Calculated Gibbs free energy change (**ΔG**) of each active site as labeled in **b**.

Supplementary Tables.

Supplementary Table 1. Elemental analysis of the COFs.

COFs	C wt.%	N wt.%	H wt.%
***g*-$C_{40}N_3$-COF**	84.68 (87.89)	8.38 (7.69)	4.95 (4.43)
***g*-$C_{31}N_3$-COF**	80.34 (86.09)	10.54 (9.71)	5.16 (4.20)
***g*-$C_{37}N_3$-COF**	86.88 (87.55)	8.11 (8.28)	4.26 (4.17)

The values in brackets are calculational elemental contents based on their repeated units.

Supplementary Table 2. Fluorescence life-times.

Samples	λ_{em}	Fluorescence Quantum yield	τ_1 / ns	I_1 / %	τ_2 / ns	I_2 / %	τ_{avg} / ns
***g*-$C_{40}N_3$-COF**	**563**	**0.7%**	**1.26**	**31.2**	**3.98**	**68.8**	**3.13**
***g*-$C_{31}N_3$-COF**	**572**	**2.0%**	**0.97**	**28.8**	**3.41**	**71.2**	**2.71**
***g*-$C_{37}N_3$-COF**	**570**	**3.2%**	**0.73**	**24.3**	**3.18**	**75.7**	**2.58**

The PL decay data were recorded upon excitation at λ_{exc} = 365 nm with a laser and observed at λ_{em} = 563, 572 and 570 nm for ***g*-$C_{40}N_3$-COF**, ***g*-$C_{31}N_3$-COF** and ***g*-$C_{37}N_3$-COF**, respectively. The life-time fittings were performed according to $y = A_0 + A_1\exp(-x/\tau_1) + A_2\exp(-x/\tau_2)$. The intensity of life-time was calculated from $I_i = A_i\tau_i / (A_1\tau_1 + A_2\tau_2)$, i=1, 2. The average life-time was calculated from $\tau_{avg} = \tau_1 I_1 + \tau_2 I_2$.

Supplementary Table 3. Hydrogen evolution rates for ***g*-$C_{40}N_3$-COF** using different sacrificial agents.

Sacrificial Agents	TEoA[a]	Sodium L-Ascorbate[b]	MeOH[a]	EtOH[a]	Na_2SO_3[b]	TEA[a]	Na_2S[b]	Lactic Acid[a]
**HER /μmol h^{-1}**	**129.8**	**23.4**	**5.2**	**2.8**	**0.7**	**0.6**	**0.2**	**0**

[a] Reaction condition: 50 mg of 3 wt% Pt modified ***g*-$C_{40}N_3$-COF** was suspended in 100 mL of deionized water and 10 mL of sacrificial agent, irradiated by a 300-W Xe lamp with cut-off filter λ > 420 nm.

[b] Reaction condition: 50 mg of 3 wt% Pt modified ***g*-$C_{40}N_3$-COF** was suspended in 110 mL of an aqueous solution of the sacrificial agent with concentration of 0.1 mol L^{-1}, irradiated by a 300-W Xe lamp with cut-off filter λ > 420 nm.

Supplementary Table 4. Fractional atomic coordinates for the eclipsed AA-stacking unit cell of *g*-$C_{40}N_3$-COF.

Space group: $P\bar{1}$

a = 37.419 Å, b = 37.843 Å, c = 3.588 Å

α = 90.044°, β = 90.061°, γ = 119.756°

Atom	*x*/*a*	*y*/*b*	*z*/*c*

N	0.18313	-0.12718	0.62216
C	0.20728	-0.14468	0.62832
C	0.25255	-0.11682	0.62136
C	0.27921	-0.12604	0.46035
H	0.26408	-0.08737	0.75327
H	0.26827	-0.1546	0.31572
C	0.34911	-0.11703	0.52452
H	0.33534	-0.1497	0.55514
C	0.39191	-0.09281	0.53684
H	0.41006	-0.10747	0.58311
C	0.41082	-0.05032	0.49432
C	0.38562	-0.03275	0.44538
H	0.39896	-0.00035	0.40173
C	0.34275	-0.05696	0.44183
H	0.32419	-0.04268	0.39795
C	0.32422	-0.09932	0.48036
C	0.47561	0.01526	0.64194
H	0.45756	0.02745	0.76396
C	-0.48156	0.03946	0.64411
H	-0.46811	0.06945	0.76792
C	-0.45653	0.02455	0.50222
C	0.12144	-0.19163	0.6768
C	0.14141	-0.14886	0.64774
C	0.11731	-0.12739	0.65282
C	0.13084	-0.0901	0.51312
H	0.08682	-0.14309	0.77302
H	0.16196	-0.07278	0.40697
C	0.1211	-0.02951	0.53627
H	0.15396	-0.01028	0.57502
C	0.0959	-0.01186	0.53638
H	0.11006	0.02062	0.58091
C	0.05298	-0.03601	0.48939
C	0.03611	-0.07815	0.43727
H	0.00349	-0.09765	0.38814
C	0.0612	-0.09577	0.44207
H	0.04707	-0.12825	0.39613
C	0.10391	-0.0717	0.49832
C	-0.01435	-0.03919	0.6388
H	-0.0259	-0.06915	0.7584
C	-0.03983	-0.02195	0.64375
H	-0.07008	-0.03918	0.76708
C	-0.02587	0.01751	0.50506
C	0.07735	-0.21439	0.7379

N	0.04216	-0.23242	0.78644
C	0.18849	-0.18757	0.66443
C	0.14505	-0.21149	0.66597
C	0.12558	-0.25685	0.67063
C	0.09102	-0.28188	0.48525
H	0.14098	-0.27015	0.82312
H	0.07494	-0.26998	0.32457
C	0.03048	-0.35113	0.52939
H	0.01106	-0.3376	0.562
C	0.01268	-0.39344	0.53562
H	-0.02017	-0.41157	0.57997
C	0.03709	-0.41191	0.49183
C	0.07984	-0.38681	0.44399
H	0.09952	-0.3998	0.39808
C	0.09771	-0.34447	0.44705
H	0.1306	-0.32587	0.40579
C	0.0731	-0.32639	0.49141
C	0.03964	-0.47559	0.64159
H	0.06989	-0.45749	0.76349
C	0.0218	0.48208	0.64603
H	0.03893	0.46902	0.77125
C	-0.01809	0.45705	0.50439
C	0.21294	-0.20711	0.72314
N	0.23229	-0.22292	0.77086

Supplementary Table 5. Fractional atomic coordinates for the eclipsed AA-stacking unit cell of *g*-$C_{31}N_3$-COF.

Space group: *P*2

a = 30.365 Å, *b* = 3.573 Å, *c* = 30.312 Å

α = 90.000°, *β* = 58.819°, *γ* = 90.000°

Atom	*x*/*a*	*y*/*b*	*z*/*c*
N	1.18708	-0.58277	-0.805
C	1.21788	-0.58973	-0.8572
C	1.27425	-0.58747	-0.87863
C	1.30811	-0.42095	-0.92308
H	1.28817	-0.72442	-0.85625
H	1.29538	-0.27131	-0.94539
C	1.39653	-0.52016	-0.99676
H	1.38068	-0.58435	-1.02072
C	1.44977	-0.52977	-1.01834
H	1.47363	-0.60947	-1.05841
C	1.47155	-0.44262	-0.98835
C	1.43838	-0.35228	-0.93593

H	1.45344	-0.27097	-0.91189
C	1.38506	-0.35476	-0.91405
H	1.36042	-0.28039	-0.87383
C	1.3639	-0.4373	-0.94444
C	0.80489	-0.61831	-0.11221
C	0.77361	-0.67342	-0.05693
N	0.74887	-0.71776	-0.01276
C	1.11061	-0.62391	-0.80911
C	1.13491	-0.60098	-0.78025
C	1.10406	-0.60648	-0.72322
C	1.11934	-0.45095	-0.6933
H	1.06659	-0.73866	-0.70475
H	1.15724	-0.32656	-0.71009
C	1.10345	-0.51917	-0.60451
H	1.14369	-0.58818	-0.62082
C	1.0703	-0.52485	-0.551
H	1.08593	-0.60515	-0.52743
C	1.01766	-0.43737	-0.52856
C	0.99919	-0.34766	-0.56131
H	0.95946	-0.26796	-0.54587
C	1.03204	-0.35261	-0.61475
H	1.01659	-0.28222	-0.63886
C	1.08444	-0.44003	-0.63678
C	1.05536	-0.67712	-0.78245
N	1.01121	-0.71929	-0.7609
C	1.14084	-0.61387	-0.86366
C	1.11738	-0.61526	-0.89656
C	1.07416	-0.43237	-0.88481
H	1.13734	-0.76441	-0.93287
H	1.05319	-0.27236	-0.84964
C	1.00109	-0.52017	-0.89843
H	0.97665	-0.58636	-0.85786
C	0.98043	-0.5272	-0.93025
H	0.94051	-0.60679	-0.91338
C	1.01115	-0.43954	-0.98308
C	1.06338	-0.35042	-1.00316
H	1.08806	-0.26966	-1.04307
C	1.08432	-0.35508	-0.97158
H	1.12447	-0.28298	-0.98785
C	1.05323	-0.43952	-0.91901

Supplementary Table 6. Fractional atomic coordinates for the eclipsed AA-stacking unit cell of *g*-$C_{37}N_3$-COF.

Space group: *P*1

a = 19.010 Å, *b* = 18.969 Å, *c* = 3.749 Å

α = 90.295°, *β* = 90.092°, *γ* = 120.818°

Atom	x/a	y/b	z/c
C	0.08662	0.04818	0.43584
C	0.03961	0.08614	0.43567
H	0.07022	0.15225	0.43484
C	0.22055	0.17735	0.2894
H	0.18818	0.20182	0.148
C	0.30601	0.22528	0.30792
H	0.33643	0.28557	0.18784
C	0.35193	0.19552	0.47256
C	0.30986	0.11561	0.61419
H	0.3413	0.08827	0.73837
C	0.22453	0.06863	0.60381
H	0.19532	0.00957	0.73349
C	0.17822	0.09855	0.44181
C	0.4428	0.24904	0.48424
C	0.49222	0.22973	0.65818
H	0.47029	0.3058	0.33511
H	0.46701	0.17409	0.81066
N	0.61902	0.36386	0.62534
C	0.58258	0.2809	0.64496
C	0.95352	0.03975	0.43905
C	0.91443	0.95363	0.44077
H	0.8484	0.91743	0.44315
C	0.82617	0.04374	0.28597
H	0.80092	0.98582	0.14503
C	0.77985	0.08179	0.29714
H	0.71983	0.05124	0.17253
C	0.81068	0.15874	0.46135
C	0.88972	0.1974	0.61212
H	0.91694	0.25723	0.73643
C	0.93542	0.15861	0.60758
H	0.99425	0.18894	0.7413
C	0.90451	0.08116	0.44408
C	0.75857	0.19603	0.46265
C	0.77207	0.26017	0.66604
H	0.70595	0.16866	0.28615
H	0.82523	0.29005	0.83766
C	0.63128	0.24473	0.66655

C	0.71762	0.29534	0.6576
C	0.59247	0.1567	0.71241
N	0.56134	0.08646	0.74886
C	0.95962	0.91373	0.44016
C	0.04565	0.9619	0.4372
H	0.08097	0.93197	0.43726
C	0.95344	0.78103	0.28366
H	0.01125	0.81435	0.1455
C	0.9143	0.69559	0.28853
H	0.9442	0.66629	0.16338
C	0.83716	0.64817	0.44634
C	0.79999	0.68854	0.60259
H	0.74075	0.65503	0.7282
C	0.83991	0.77427	0.60394
H	0.81064	0.80276	0.74043
C	0.91718	0.82203	0.44293
C	0.79925	0.55763	0.43979
C	0.73301	0.50449	0.62574
H	0.82878	0.5336	0.27402
H	0.69883	0.52528	0.77815
C	0.75301	0.38149	0.65548
C	0.7016	0.41504	0.62807
C	0.84085	0.43413	0.69929
N	0.911	0.47598	0.7349

Supplementary Table 7. Photocatalytic H_2 evolution performance of the reported COF photocatalysts.

Photocatalyst	Cocatalyst	AQY (%)	Sacrificial agent	Reference
***g*-$C_{40}N_3$-COF**	**3 wt% Pt**	**4.84 (λ = 420 nm)**	**TEoA**	**This work**
TFPT-COF	2.2 wt% Pt	2.2 (λ = 400 nm)	TEoA	[4]
N_3-COF	3 wt% Pt	0.44 (λ = 450 nm)	TEoA	[5]
TP-BDDA	3 wt% Pt	1.3 (λ = 420 nm)	TEoA	[6]
FS-COF	3 wt% H_2PtCl_6	3.2 (λ = 420 nm)	ascorbic acid	[7]

Supplementary Table 8. The calculated Gibbs free energy changes (**ΔG**, eV) for intermediates at different active sites of HER on ***g*-$C_{40}N_3$-COF** at pH 10.30.

Site	N1	N2	N3	C1	C2	C3	C4
**ΔG_A (eV)**	0.23691	-0.3564	0.23233	0.95092	0.17233	0.16611	1.03848
**ΔG_B (eV)**	-1.06531	-0.472	-1.06073	-1.77932	-1.00073	-0.99451	-1.86688

Site	C5	C6	C7	C8	C9	C10	C11
**ΔG_A (eV)**	0.0597	0.28624	0.95906	0.54394	0.64504	0.66742	0.62037
**ΔG_B (eV)**	-0.8881	-1.11464	-1.78746	-1.37234	-1.47344	-1.49582	-1.44877

Site	C12	C13	C14	C15
**ΔG_A (eV)**	0.94692	0.62651	0.42732	0.71605
**ΔG_B (eV)**	-1.77532	-1.45491	-1.25572	-1.54445

Supplementary References

1. Wang, H. et al. Synthesis and properties of new two-photon absorption chromophores containing 3,5-dicyano-2,4,6-tristyrylpyridine as the core. *New J. Chem.* **29**, 792-797 (2005).
2. Modak, A., Nandi, M., Mondal, J. & Bhaumik, A. Porphyrin based porous organic polymers: novel synthetic strategy and exceptionally high CO_2 adsorption capacity. *Chem. Commun.* **48**, 248-250 (2012).
3. Wang Z. et al. Phenanthro[9,10-*d*]imidazole as a new building block for blue light emitting materials. *J. Mater. Chem.* **21**, 5451-5456 (2011).
4. Stegbauer, L., Schwinghammer, K. & Lotsch, B. V. A hydrazone-based covalent organic framework for photocatalytic hydrogen production. *Chem. Sci.* **5**, 2789-2793 (2014).
5. Vyas, V. S. et al. A tunable azine covalent organic framework platform for visible light-induced hydrogen generation. *Nat. Commun.* **6**, 8508 (2015).
6. Pachfule, P. et al. Diacetylene functionalized covalent organic framework (COF) for photocatalytic hydrogen generation. *J. Am. Chem. Soc.* **140**, 1423-1427 (2018).
7. Wang, X. et al. Sulfone-containing covalent organic frameworks for photocatalytic hydrogen evolution from water. *Nat. Chem.* **10**, 1180-1189 (2018).
8. Nørskov J. K. et al. Origin of the overpotential for oxygen reduction at a fuel-cell cathode. *J. Phys. Chem. B.* **108**, 17886–17892 (2004).
